# Characterization of phytochemicals from twisted-leaf garlic (*Allium obliquum* L.) using liquid chromatography coupled with electrospray ionization quadrupole time-of-flight mass spectrometry

**DOI:** 10.1007/s11306-023-02054-2

**Published:** 2023-10-21

**Authors:** Christoph Böttcher, Linh T. Bach, Melanie Stürtz, Hartwig Schulz

**Affiliations:** 1 Federal Research Centre for Cultivated Plants, Institute for Ecological Chemistry, Plant Analysis and Stored Product Protection, Julius Kühn Institute (JKI), Königin-Luise-Strasse 19, 14195 Berlin, Germany; 2 Symrise AG, Mühlenfeldstrasse 1, 37603 Holzminden, Germany; 3 Consulting and Project Management for Medicinal and Aromatic Plants, Waltraudstrasse 4, 14532 Stahnsdorf, Germany

**Keywords:** *Allium obliquum*, Metabolomics, Tandem mass spectrometry, Fructooligosaccharides, Alkenylcysteine sulfoxides, Flavonoids, Hydroxycinnamic acid conjugates, Steroidal saponins

## Abstract

**Introduction:**

Twisted-leaf garlic (*Allium obliquum* L.) is a wild *Allium* species, which is traditionally used as aroma plant for culinary purposes due to its unique, garlic-like flavor. It represents an interesting candidate for domestication, breeding and cultivation.

**Objectives:**

The objective of this work was to explore and comprehensively characterize polar and semi-polar phytochemicals accumulating in leaves and bulbs of *A. obliquum*.

**Method:**

Plant material obtained from a multiyear field trial was analyzed using a metabolite profiling workflow based on ultra-high performance liquid chromatography-coupled electrospray ionization quadrupole time-of-flight mass spectrometry (UHPLC/ESI-QTOFMS) and two chromatographic methods. For annotation of metabolites, tandem mass spectrometry experiments were carried out and the resulting accurate-mass collision-induced dissociation (CID) mass spectra interpreted. Onion and garlic bulb extracts were used as reference samples.

**Results:**

Important metabolite classes influencing nutritional, sensory and technological properties were detected and structurally characterized including fructooligosaccharides with a degree of polymerization of 3–5, *S*-alk(en)ylcysteine sulfoxides and other *S*-substituted cysteine conjugates, flavonoids including *O*- and *C*-glycosylated flavones as well as *O*-glycosylated flavonols, steroidal saponins, hydroxycinnamic acid conjugates, phenylethanoids and free sphingoid bases. In addition, quantitative data for non-structural carbohydrates, *S*-alk(en)ylcysteine sulfoxides and flavonoids are provided.

**Conclusion:**

The compiled analytical data including CID mass spectra of more than 160 annotated metabolites provide for the first time a phytochemical inventory of *A. obliquum* and lay the foundation for its further use as aroma plant in food industry.

**Supplementary Information:**

The online version contains supplementary material available at 10.1007/s11306-023-02054-2.

## Introduction

Within the monocotyledons *Allium* is one of the largest genera and comprises about 1000 species which are naturally distributed in temperate and subtropical zones of the Northern Hemisphere (Govaerts et al., 2023). Because of their outstanding flavoring and health-promoting properties, several *Allium* species are used for culinary purposes or as medicinal plants. Today, about 25 *Allium* species are cultivated (Fritsch & Friesen, 2002). Onion (*A. cepa* L. cepa group), shallot (*A. cepa* L. aggregatum group) and garlic (*A. sativum* L.) represent the economically most important species, which are grown and consumed almost worldwide. Other cultivated *Allium* species such as leek (*A. ampeloprasum* L. leek group), bunching onion (*A. fistulosum* L.) or rakkyo (*A. chinense* G. Don) are of regional importance as vegetables and aromatic plants.

Numerous wild *Allium* species possess similar odor and taste characteristics as cultivated ones and are traditionally collected or grown in kitchen gardens by the local population. Among them twisted-leaf garlic (*Allium obliquum* L.), also referred to as “oblique onion” or “lop-sided onion”, is characterized by a unique, intensive aroma with garlic-like notes. In particular, its bulbs are therefore locally used as a garlic substitute (Seidemann, 2005). The main geographic range of *A. obliquum* lies in Central Asia (Chuhina, 2008; Friesen et al., 2021) with a disjunct distribution in mountain ranges and partially in surrounding lowlands and plains in Russia, Kazakhstan, Mongolia and China (e.g. Southern Urals, Altai, West Sayan, Saur-Tarbagatai). However, isolated habitats can also be found in Europe, e.g. in Romania, in Ukraine and in the southeast of European Russia (Didukh et al., 1982). In connection with its occurrence in the Turda Gorge in Romania the traditional use of *A. obliquum* as garlic substitute for culinary purposes was documented by the German pharmacist and botanist Gabriel Wolff in the middle of the nineteenth century (Wolff, 1865). Due to excessive collecting by the local population in the past, *A. obliquum* represents nowadays a critically endangered species in Romania and was included in the IUCN Red List (Kell et al., 2011). *A. obliquum* was also listed as a wild food plant used by Hungarians in the Carpathian Basin during the nineteenth and twentieth centuries (Denes et al., 2012).

*Allium obliquum* is a herbaceous, perennial plant usually producing one, rarely up to three bulbs (Chuhina, 2008; Friesen et al., 2021). The bulbs have an oblong-ovoid shape and leathery reddish brown outer tunics. Depending on their age, they attain diameters of 1–3 cm and weights of 5–20 g. Leaf sheaths cover two thirds of the stem, which is typically 70–130 cm tall. The leaves are linear, flat, 15–40 cm long, at the base 7–20 mm wide and gradually narrowing to the tip. The inflorescence has a globose dense shape with many yellow-green flowers crowded together. As a mesophyte the photophilous plant prefers habitats such as meadows, wooded mountain slopes, banks of mountain rivers and rock ledges. Depending on the individual location, the flowering period lasts from May to August. Based on sequence data of the internal transcribed spacer region of nuclear ribosomal DNA, *A. obliquum* was phylogenetically classified to the subgenus Polyprason, section Oreiprason (Friesen et al., 2006).

In contrast to economically important species of the genus *Allium* the phytochemistry of *A. obliquum* is less explored. Sporadic phytochemical analyses are currently limited to alk(en)ylcysteine sulfoxides (ACSO) and their degradation products as well as polyphenolic compounds. In accordance with its garlic-like aroma alliin represents the quantitatively predominating ACSO in *A. obliquum*. Krest et al. exclusively detected alliin and methiin in bulbs of *A. obliquum* in a mass ratio of 56/44 (Krest et al., 2000), whereas two other studies reported the presence of alliin, methiin and minor amounts of propiin and isoalliin in a mass ratio of 57/30/9/4 (Fritsch & Keusgen, 2006; Keusgen et al., 2002). Published total amounts of ACSO in bulbs vary by more than one order of magnitude ranging from 0.64 mg/g fresh weight (Krest et al., 2000) to 8.8 mg/g fresh weight (Fritsch & Keusgen, 2006) and 13.4 mg/g fresh weight (Keusgen et al., 2002). The profile of volatile sulfur compounds of homogenized and enzymatically autolyzed bulb tissue was determined by headspace solid-phase microextraction gas chromatography mass spectrometry (Keusgen et al., 2002). In accordance with the relative abundances of the corresponding amino acid precursors di-2-propenyl disulfide (81.3%), methyl 2-propenyl disulfide (5.7%), (*E*)-1-propenyl-2-propenyl disulfide (4.9%), di-2-propenyl sulfide (4.3%), 2-propenyl-propyl-disulfide (1.0%) were detected as major components. Three hydroxycinnamic acids (*p*-coumaric, ferulic and sinapic acid), two flavones (apigenin and luteolin) and two flavonols (kaempferol and quercetin) were identified in hydroethanolic extracts of leaves and stems of *A. obliquum* after acidic hydrolysis (Vlase et al., 2012).

Due to the formation of comparatively large plants with bulbs as storage organs, the intensive garlic-like aroma and ethnobotanical use as aroma plant, *A. obliquum* represents an interesting species for cultivation, domestication and hybridization with other *Allium* species such as *A. cepa* (Keusgen et al., 2002). In addition, various technological methods have been recently developed to obtain promising new flavor extracts from the bulbs of *A. obliquum* (Stürtz et al., 2022a, 2022b). Within the frame of a multi-year cultivation trial, we were interested in a more detailed characterization of phytochemicals accumulating in bulbs and leaves of *A. obliquum*. As the main analytical tool, an adapted metabolomics workflow based on ultra-high performance liquid chromatography-coupled electrospray ionization quadrupole time-of-flight mass spectrometry (UHPLC/ESI-QTOFMS) was applied. The workflow was originally developed in our laboratory for comprehensive characterization of phytochemicals from onion bulbs and covers important metabolite classes, which influence their nutritional, sensory and technological properties (Böttcher et al., 2017).

The following manuscript presents a mass spectrometry-based characterization of the main phytochemicals of *A. obliquum* including fructooligosaccharides (FOS), *S*-substituted cysteine conjugates, flavonoids, steroidal saponins and hydroxycinnamic acid conjugates and provides chromatographic and tandem mass spectral data of the annotated compounds. Quantitative data of FOS, ACSO and flavonoids using plant material from three cultivation years complement data from the qualitative screening and together provide for the first time a phytochemical inventory of *A. obliquum*.

## Materials and methods

### Plant material

*Allium obliquum* L. (accession number All 1221) was obtained from the Federal ex situ Gene Bank of the Leibniz Institute of Plant Genetics and Crop Plant Research (IPK) at Gatersleben, Germany. After an initial propagation phase, plants were grown from sets at the experimental station of the Julius Kühn Institute in Berlin-Dahlem (52.45943 N, 13.29916 E, 45 m a.s.l., mean annual temperature 8.8 °C, mean annual precipitation 580 mm, loamy sand (75% sand, 17% silt, 8% clay), pH 6.2, 2.1% soil organic matter, C/N 11) in 2016, 2017 and 2018. The sets were planted on March 14, 2016 at 7.5 cm spacing in rows 30 cm apart on a plot of 1.5 m × 30 m. Fertilization, irrigation and plant protection was based on the practice of commercial onion cultivation. Twenty well-developed, healthy plants without scape and inflorescence were harvested each on June 1, 2016, on May 16, 2017 and on June 6, 2018 for phytochemical analyses. Intact young garlic plants (*Allium sativum* L., accession number All 1318) were obtained from the Federal ex situ Gene Bank of the IPK on May 17, 2017. Onion plants (*Allium cepa* L., cultivar ´Sturon´) were harvested on June 26, 2018 from a commercial field near Holzminden, Germany.

### Chemicals

Ethanol (≥ 99.9%, for HPLC), methanol (≥ 99.95%, for LC–MS) and acetonitrile (≥ 99.95%, for LC–MS), ethyl acetate (≥ 99.8%, for residue analysis), n-hexane (≥ 95%, for residue analysis) and acetic acid (≥ 99.5%, p.a.) were purchased from Th. Geyer GmbH (CHEMSOLUTE). Ultra-pure water (resistivity ≥ 18.2 MΩ cm) was obtained from a water purification system (Arium 611, Sartorius). Formic acid (≥ 98%, for LC–MS), sodium acetate (puriss. p.a.) and Diaion HP-20 polymeric adsorbent were supplied by Sigma-Aldrich. Inulinase (EC 3.2.1.7, from *Aspergillus niger*, lyophilized powder, 25 units mg^−1^), β-glucuronidase (EC 3.2.1.31, from limpets, aqueous solution, 100 units µL^−1^) and β-glucosidase (EC 3.2.1.21, from almonds, lyophilized powder, 6.7 units mg^−1^) were purchased from Sigma-Aldrich. Sources of reference compounds used for authentication of metabolites or calibration standards are listed in Supplemental Table 1.

### Processing of plant material

Freshly harvested plants were cleaned of adhering soil. Dry leaves were removed and roots were cut off along the basal plate. Afterwards, the chlorophyll-free part of the plants containing the developing bulb was separated and the remaining aerial part was cut into 15 cm long pieces. Plant material was vacuum packed, immediately frozen at − 80 °C and stored at the same temperature until freeze drying. Deep-frozen plant material was dried to constant weight for 14 days using a freeze dryer (Gamma 1–16 LSC, Christ, condenser temperature − 50 °C, pressure 0.04 mbar). Dried plant material was cut into 1 cm long pieces, homogenized batch-wise using a knife mill (Tube mill control, IKA, 5000 min^−1^ for 60 s, then 25,000 min^−1^ for 90 s) and stored at − 80 °C until extraction.

### Extract preparation for metabolite profiling

Freeze-dried and homogenized plant material (200 mg ± 5 mg) was weighed into a 15 mL centrifuge tube and 5 mL methanol/water, 8/2 (v/v) were added. The mixture was vortex-mixed (30 s), sonicated (5 min, 22–25 °C) and shaken (30 min, 2400 min^−1^, 22 °C). After centrifugation (4696×*g*, 10 min, 22 °C) the supernatant was transferred into a 10 mL volumetric flask. The remaining pellet was extracted once again with 4.5 mL methanol/water, 8/2 (v/v) following the above described procedure. After centrifugation both supernatants were combined and their volume adjusted to 10 mL using methanol/water, 8/2 (v/v). Corresponding aliquots of the stock extract were diluted with water (1/20 dilution for profiling of saccharides and *S*-substituted cysteine conjugates using chromatographic method A, 1/2 dilution for profiling of semi-polar metabolites using chromatographic method B) and centrifuged (13,000×*g*, 10 min, 22 °C). The resulting supernatants were transferred into vials and stored in a fridge at 6 °C until analysis.

### UHPLC/DAD/ESI-QTOFMS

LC/MS analyses were performed on an Infinity 1290 series UHPLC system (Agilent Technologies) consisting of a binary pump (G4220A), an autosampler (G4226A, 20 µL loop), an autosampler thermostat (G1330B), a thermostatted column compartment (G1316C) which was coupled in series with a diode array detector (G4212B) and an iFunnel Q-TOF mass spectrometer (G6550A, Agilent Technologies) via a dual Agilent jet stream electrospray ion source. MassHunter LC/MS Data Acquisition software (version B.06.01) was used for controlling the instrument and data acquisition as well as MassHunter Qualitative and Quantitative Analysis software (version B.07.00) for data evaluation. The mass spectrometer was operated in low mass range (*m/z* 1700) and extended dynamic range (2 GHz) mode. Using these settings, the mass resolution (full width at half maximum) at *m/z* 922 was approx. 23,000. The instrument was auto tuned and calibrated according to manufacturer´s recommendations using ESI-L tuning mix (Agilent Technologies). Reference mass correction was used throughout all experiments. For this purpose, a solution of purine (20 µM) and hexakis-(2,2,3,3-tetrafluoropropoxy)phosphazine (20 µM) in acetonitrile/water, 95/5 (v/v) was continuously introduced through the second sprayer of the dual ion source at a flow rate of 20 µL min^−1^ using an external HPLC pump equipped with a 1:100 splitting device.

#### Chromatographic method A

Extracts (500 nL injection volume) were separated on a Develosil RP-Aqueous C30 column (3.0 mm × 150 mm, 3 µm particle size, 140 Å pore size, Phenomenex) using 0.25% (v/v) formic acid in water and 0.25% (v/v) formic acid in methanol as eluent A and B, respectively. The following binary gradient program at a flow rate of 500 µL min^−1^ was applied: 0–13 min, linear from 0 to 9.75% B; 13–17 min, isocratic, 95% B; 17–20 min, isocratic, 0% B. The column temperature was maintained at 40 °C and the autosampler temperature at 6 °C. Eluting compounds were detected in an *m/z* range of 70–1700 either in positive or negative ion mode. Mass spectra were acquired in centroid mode using an acquisition rate of 2 spectra per second. For further instrument settings and parameters, see Böttcher et al. (2017).

#### Chromatographic method B

Extracts (1 µL) were separated on a Zorbax RRHD Eclipse Plus C18 column (2.1 mm × 100 mm, 1.8 µm particle size, Agilent Technologies) using 0.1% (v/v) formic acid in water and 0.1% (v/v) formic acid in acetonitrile as eluent A and B, respectively. The following binary gradient program at a flow rate of 400 µL min^−1^ was applied: 0–20 min, linear from 5 to 65% B; 20–23 min, isocratic, 95% B; 23–25 min, isocratic, 5% B. The column temperature was maintained at 40 °C and the autosampler temperature at 6 °C. Eluting compounds were sequentially detected in a wavelength range of 190–600 nm and in an *m/z* range of 70–1700 either in positive or negative ion mode. Absorption spectra were acquired using an acquisition rate of 2.5 spectra per second, centroid mass spectra using an acquisition rate of 3 spectra per second. Instrument settings were as described in method A.

#### Acquisition of collision-induced dissociation (CID) mass spectra

CID mass spectra were acquired in targeted-MS^2^ mode using scheduled precursor ion lists and the following parameters: acquisition rate MS, 3 spectra per second; acquisition rate MS/MS, 3 spectra per second, isolation width, narrow (1.3 m*/z*); collision energy, 5—60 V; collision gas, nitrogen. For acquisition of CID mass spectra of in-source fragment ions (pseudo-MS^3^) funnel exit DC voltage was increased from 50 to 120 V. For all-ion fragmentation experiments, collision energy was alternated in MS mode between 0 and 10 V, 0 and 20 V or 0 and 30 V.

### Quantification of non-structural carbohydrates

#### Extraction

Freeze-dried and homogenized plant material (200 mg ± 5 mg) was weighed into a 15 mL centrifuge tube. After addition of 3 mL ethanol/water, 1/1 (v/v) the mixture was vortex-mixed (1 min) and the resulting suspension shaken (10 min, 990 min^−1^, 80 °C). After centrifugation (10 min, 3000×*g*, 22 °C) the supernatant was transferred into a 10 mL volumetric flask. The remaining pellet was extracted twice more with 3 mL ethanol/water, 1/1 (v/v) following the above described procedure. All three extracts were combined and their volume was adjusted to 10 mL using ethanol/water, 1/1 (v/v).

#### Quantification of free glucose, fructose and sucrose

One thousand microliter of the stock extract were transferred into a 2 mL microcentrifuge tube and evaporated to dryness using a centrifugal vacuum concentrator (60 °C, 10 mbar). Four hundred microliter acetonitrile/water, 1/1 (v/v) were added to the remaining pellet. The mixture was sonicated (5 min, 22 °C), shaken (10 min, 2400 min^−1^, 22 °C) and centrifuged (10 min, 13,000×*g*, 22 °C). An aliquot of the supernatant was subjected to HPLC coupled with evaporative light scattering detection (HPLC/ELSD). Free glucose (Glc^free^), free fructose (Fru^free^) and sucrose (Suc) were quantified using external calibrations as previously described (Krähmer et al., 2021).

#### Quantification of total glucose and fructose

One thousand microliter of the stock extract were transferred into a 2 mL microcentrifuge tube and evaporated to dryness using a centrifugal vacuum concentrator (60 °C, 10 mbar). The remaining pellet was dissolved in 60 µL water and 40 µL of a freshly prepared inulinase solution (100 units mL^−1^ in water) were added. The solution was shaken (60 min, 990 min^−1^, 57 °C). After cooling to room temperature, it was diluted with 900 µL water, quantitatively transferred into a 2 mL volumetric flask and diluted with acetonitrile to a final volume of 2 mL. The solution was centrifuged (10 min, 13,000×*g*, 22 °C) and an aliquot of the resulting supernatant was subjected to HPLC/ELSD for quantification of total glucose (Glc^total^) and total fructose (Fru^total^). To verify completeness of enzymatic hydrolysis, another aliquot was subjected to LC/MS (chromatographic method A, negative ion mode) for analysis of FOS.

The total concentration of fructooligosaccharides (FOS) was calculated according to the literature (Prosky & Hoebregs, 1999) using the following equations:

$$\left[ {Glc^{FOS} } \right] = \left[ {Glc^{total} } \right] - \left[ {Glc^{free} } \right] - \frac{1}{1.9} \left[ {Suc} \right]$$

$$\left[ {Fru^{FOS} } \right] = \left[ {Fru^{total} } \right] - \left[ {Fru^{free} } \right] - \frac{1}{1.9}\left[ {Suc} \right]$$

$$\left[FOS\right]=0.91 (\left[{Glc}^{FOS}\right]+\left[{Fru}^{FOS}\right])$$

Reported concentrations represent averages of two technical replicates.

### Quantification of alk(en)ylcysteine sulfoxides

Diluted stock extracts (1/20, concentration of 1 mg dry weight per mL extract, see paragraph 2.4) were used for quantification of ACSO. A stock solution containing l-( +)-methiin and l-( +)-alliin at a concentration of 1 mM was prepared in water and serially diluted with water. Extracts and calibration samples were analysed using chromatographic method A in positive ion mode. Target metabolites were quantified by integration of extracted ion chromatograms generated for protonated molecular ions (Supplemental Table 4a). Calibration curves were established for methiin (calibration range 2–50 pmol on column, 5 points in duplicate, quadratic regression model, weigthing 1/x, R^2^ = 0.9976) and alliin (calibration range 5–100 pmol, 5 points in duplicate, quadratic regression model, weigthing 1/x, R^2^ = 0.9996; calibration range 0.2–5 pmol, 5 points in duplicate, quadratic regression model, weigthing 1/x, R^2^ = 0.9988). Due to the lack of reference compounds and the structural similarity to alliin, other ACSO including isoalliin, cycloalliin and propiin were quantified using the calibration curve of alliin. Reported concentrations represent averages of two technical replicates.

### Enzymatic hydrolysis of flavonoid glycosides and quantification of released aglycones

An aliquot (200 µL) of a hydromethanolic leaf extract (concentration of 20 mg dry weight per mL extract, see paragraph 2.4) was transferred into a 2 mL microcentrifuge tube and evaporated to dryness using a centrifugal vacuum concentrator (35 °C, 10 mbar). To the remaining residue 290 µL sodium acetate buffer (50 mM, pH 5.0), 10 µL β-glucuronidase solution (100 units µL^−1^) and 100 µL freshly prepared β-glucosidase solution (0.134 units µL^−1^, in 50 mM sodium acetate buffer, pH 5.0) were added. The mixture was vortex-mixed (1 min) and shaken (22 h, 990 min^−1^, 40 °C). Afterwards the reaction mixture was extracted three times with 400 µL ethyl acetate. The combined organic extracts were evaporated to dryness using a centrifugal vacuum concentrator (35 °C, 10 mbar) and 1000 µL methanol/water, 4/1 (v/v) were added to the remaining pellet. The resulting mixture was shaken (10 min, 2400 min^−1^, 22 °C), centrifuged (10 min, 13,000×*g*, 22 °C) and the resulting supernatant transferred into an HPLC vial.

Individual stock solutions of apigenin, luteolin, chrysoeriol, tricin, kaempferol, quercetin and isorhamnetin at a concentration of 2.5 mM were prepared in methanol/acetone, 1/1 (v/v). Using these stock solution, a mixture containing each flavonoid at a concentration of 50 µM was prepared in methanol/water, 1/1 (v/v) and serially diluted with methanol/water, 1/1 (v/v). Extracts and calibration samples were analysed using chromatographic method B in negative ion mode. Target metabolites were quantified by integration of extracted ion chromatograms generated for deprotonated molecular ions. Calibration curves were established for apigenin and luteolin (calibration range 0.125–12.5 pmol on column, 7 points in duplicate, quadratic regression model, weigthing 1/x, R^2^ > 0.998), chrysoeriol and tricin (calibration range 0.125–5.0 pmol on column, 6 points in duplicate, quadratic regression model, weigthing 1/x, R^2^ > 0.997) and kaempferol, quercetin and isorhamnetin (calibration range 0.125–2.5 pmol on column, 5 points in duplicate, quadratic regression model, weigthing 1/x, R^2^ > 0.997). Selgin was quantified using the calibration curve of chrysoeriol. Reported concentrations represent averages of two technical replicates.

### Isolation and structural characterization of flavone glycosides

A hydromethanolic crude extract of *A. obliquum* leaves was pre-fractionated on Diaion HP-20 using a methanol/water step gradient. Two fractions containing flavonoids were further fractionated by semi-preparative reversed-phase HPLC. This afforded 9.3 mg **32**, 2.5 mg **58** and 0.5 mg **92** which were characterized by one and two-dimensional NMR spectroscopy and sugar analysis. The detailed procedures and analytical data can be found in Supplemental Method 1.

### Isolation and enzymatic hydrolysis of the major furostanol saponin

A hydromethanolic crude extract of *A. obliquum* bulbs was fractionated by semi-preparative reversed-phase HPLC. The isolated furostanol saponin **124** was treated with β-glucosidase for 24 h at pH 5.0 and 40 °C. Following workup, hydrolysis products were analyzed by UHPLC/ESI-QTOFMS. The detailed procedure is given in Supplemental Method 2.

## Results and discussion

In order to sample both leaf and bulb material for metabolite analyses, plants were harvested in the end of the vegetative growth phase before bolting, which is typically reached under field conditions from mid-May to early June. At this stage, leaves are fully developed and bulbs reach approximately 50% of their final diameter while the flower stem is not yet visible. Twenty individual plants of the same developmental stage were harvested and pooled to give a representative leaf and bulb sample for metabolite analysis. To estimate the impact of environmental factors on metabolite levels sampling was carried out over three growing periods (June 2016, May 2017 and June 2018). Developmental stages of *A. obliquum* over the growing season 2017 starting from a bulb in late winter are shown in Supplemental Fig. 1.

### Non-structural carbohydrates

Glucose, fructose, sucrose, fructooligosaccharides (FOS) and/or longer-chain fructans represent the predominant non-structural carbohydrates in plants of the genus *Allium* (Ernst et al., 1998). As reserve carbohydrates, they account for a large proportion of the dry matter, in particular in storage organs such as bulbs. In onion (*A. cepa*), FOS of the inulin and the *neo-*inulin series with a degree of polymerization (DP) of 3–20 are formed (Pöhnl et al., 2017; Shiomi et al., 2014). Inulin-type FOS derive from 1-kestose (1-K, β*-*Fru*f*(2 → 1)-β-Fru*f*(2 ↔ 1)-α-Glc*p*) by chain elongation with fructofuranosyl moieties via β-(2 → 1) glycosidic linkages and carry a terminal glucopyranosyl moiety. The parent compound of *neo*-inulin-type FOS is neokestose (6G-K, β-Fru*f*(2 ↔ 1)-α-Glc*p*(6 ← 2)-β-Fru*f*). Since chain elongation with fructofuranosyl moieties via β-(2 → 1) glycosidic linkages can occur in this case from two sides, n–2 isomers can be formed for a DP of n (n ≥ 3). Also in garlic (*A. sativum*), inulin and *neo*-inulin series FOS were detected (Ernst et al., 1998), but in contrast to onion, longer-chain fructans with a number-average DP of 50 predominate (Baumgartner et al., 2000). It was shown that the β-(2 → 1)-linked fructofuranosyl backbone of these fructans is decorated with β-(2 → 6)-linked fructofuranosyl side chains (Baumgartner et al., 2000).

**Fig. 1:**
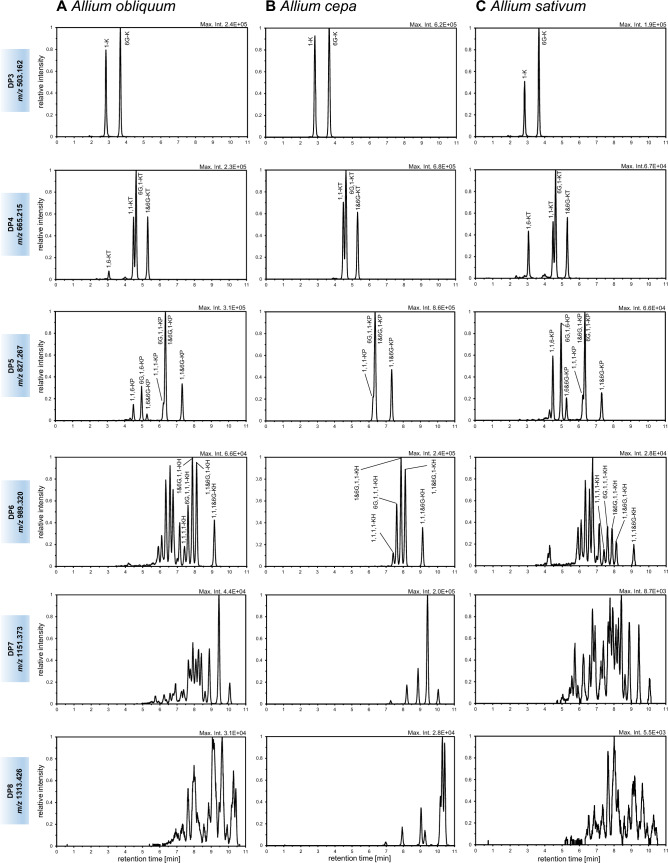
Profiles of fructooligosaccharides accumulating in bulbs of *Allium obliquum* (**A**), *Allium cepa* (**B**) and *Allium sativum* (**C**). Extracted ion chromatograms (normalized to 100%, *m/z*-width 10 ppm) of [M − H]^−^ ions of FOS with DP 3 to 8 are shown. Chromatograms were obtained using UHPLC/ESI-QTOFMS operated in negative ion mode and chromatographic method A. For schematic structures and CID mass spectra of annotated FOS, see Supplemental Figs. 2–5

The FOS composition of *A. obliquum* bulb and leaf material was analyzed using chromatographic method A. Comparison of extracted ion chromatograms (EICs) of deprotonated molecular ions of FOS with a DP of 3–8 did not reveal qualitative differences between leaf and bulb extracts. Therefore, only the FOS composition of bulbs will be discussed below. With increasing DP, FOS accumulating in bulbs of *A. obliquum* exhibited a strongly increasing isomeric complexity (Fig. 1A). For a DP of 3–6, numerous isomers were chromatographically separated, but separation gradually failed at higher DP. Elution profiles of FOS with a DP ≥ 9 therefore appeared as broad chromatographic band between 7 and 13 min.

Structural assignment of FOS isomers with a DP of 3–5 was attempted by interpretation of collision-induced dissociation (CID) mass spectra obtained from [M − H]^−^ ions and by using an onion and garlic bulb extract as reference (Fig. 1B, C). As parent compounds of the inulin and *neo*-inulin series, 1-K and 6G-K were exclusively detected as trisaccharides in bulbs of *A. obliquum* (Fig. 1A). Both kestotrioses can be differentiated on basis of their CID mass spectrum (Supplemental Fig. 2). As shown by stable isotope labelling of the fructofuranosyl moieties, CID of deprotonated 1-K resulted in formation of Z_2_^−^ and C_2_^−^ fragment ions by elimination of glucose and anhydrofructose, respectively (Harrison et al., 2012). In case of deprotonated 6G-K, a cross-ring cleavage of the glucopyranosyl moiety was observed upon CID resulting in formation of a prominent ^0,4^A_2_^−^ fragment ion (Supplemental Fig. 2) (Harrison, 2012). For a DP of 4–6, all theoretically possible FOS isomers of the inulin and *neo*-inulin series were detectable in bulbs of *A. obliquum*. Structural assignment of *neo*-inulin series FOS isomers was achieved using CID mass spectra of [M − H]^−^ ions via characteristic ^0,4^A_n_^−^ fragment ions which were detected at *m/z* [221.067 + (n–2) × 162.053] (n ≥ 2, Supplemental Figs. 3–5) and derived from cross-ring cleavage of the non-terminal glucopyranosyl moiety. In contrast, CID mass spectra of inulin series FOS primarily displayed fragment ions formed by cleavage of glycosidic bonds (Supplemental Figs. 3–5).

Beside inulin and *neo*-inulin series FOS additional FOS with a DP ≥ 4 were detectable in *A. obliquum*. Interestingly, these additional FOS were also present in bulbs of *A. sativum* but not in bulbs of *A. cepa* (Fig. 1). In addition to 1,1-kestotetraose (1,1-KT), 6G,1-KT and 1&6G-KT, an unknown KT was registered at shorter retention times. Its CID mass spectrum displayed prominent fragment ions at *m/z* 485.150 and *m/z* 341.108 formed by glycosidic bond cleavage and two fragment ions at *m/z* 233.066 and *m/z* 221.067 formed by cross-ring cleavage (Supplemental Fig. 3). As observed in the CID mass spectrum of deprotonated 6-kestose, the latter two could represent ^2,4^X_1_^−^ and a ^0,3^X_1_^−^ fragment ions associated with a terminal β-(2 → 6)-linked fructofuranosyl moiety (Harrison et al., 2012). Based on the observed CID mass spectrum and its similarity to a published one (Verspreet et al., 2017) the unknown tetrasaccharide was annotated as 1,6-KT.

At least three other kestopentaoses (KP) were detected in addition to inulin- and *neo*-inulin-type KPs. Using the characteristic cross-ring fragmentations of in 6-position glycosylated β-glucopyranosyl and β-fructofuranosyl moieties the three unknown KPs were annotated as 1,1,6-KP, 6G,1,6-KP and 1,6&6G-KP (Supplemental Fig. 4). Formation of these pentasaccharides can be rationalized by chain elongation of inulin-type (1,1-KT) and *neo*-inulin-type (6G,1-KT and 1&6G-KT) precursor saccharides with a β-fructofuranosyl moiety at the 6-position of the terminal fructofuranosyl moiety.

Based on the structure of the annotated saccharides three series of FOS are formed in *A. obliquum*. These comprise inulin- and *neo*-inulin-type FOS with exclusively β-(2 → 1) Fru*f*-Fru*f* linkages as well as mixed-type FOS with β-(2 → 1) and β-(2 → 6) Fru*f*-Fru*f* linkages. Due to the presence of two linkage motifs and hence a possible formation of branched structures isomeric complexity of mixed-type FOS increased more rapidly with increasing DP than for linear inulin- and *neo*-inulin-type FOS.

**Fig. 2:**
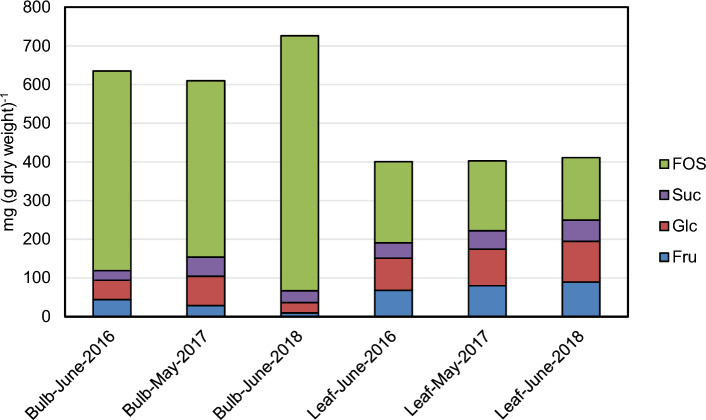
Quantification of non-structural carbohydrates in bulb and leaf tissue of *Allium obliquum*

In order to quantify non-structural carbohydrates (NSC) in bulbs and leaves of *A. obliquum* corresponding extracts were digested with inulinase and together with non-digested extracts analyzed for glucose, fructose and sucrose. Depending on the cultivation year, 61–73% of the bulb dry matter consisted of NSC (Fig. 2, Supplemental Table 2). In the physiological stage studied, a large proportion (75–91% by mass) of NSC in bulbs represented FOS with a number-average DP of 10–14 (determined from the amount of FOS-bound fructose and glucose). Consistent with the function of leaves and bulbs as source and sink organs, the amount of NSC in leaves (40–41% of dry weight) was significantly lower in comparison to bulbs. Moreover, the proportion of FOS in NSC content in leaves reached only 39–52% by mass. Accordingly, the mass fraction of monosaccharides in NSC content was significantly higher in leaves (38–47% by mass) in comparison to bulbs (5–17% by mass).

### *S*-Alk(en)ylcysteine sulfoxides and other cysteine conjugates

*S*-Alk(en)ylcysteine sulfoxides (ACSO) are the precursors of a multitude of *S*-containing compounds mediating the characteristic aroma of *Allium* plants. Upon tissue disruption, ACSO are cleaved by the vacuolar enzyme alliinase forming 2-aminoacrylate and highly reactive alk(en)yl sulfenic acids. The latter are converted in complex, mainly spontaneous follow-up reactions into sensory active compounds. The aroma type of different *Allium* species is essentially determined by a species-specific pattern of ACSO (Fritsch & Keusgen, 2006) which is dominated by alliin in garlic and isoalliin in onion.

**Fig. 3:**
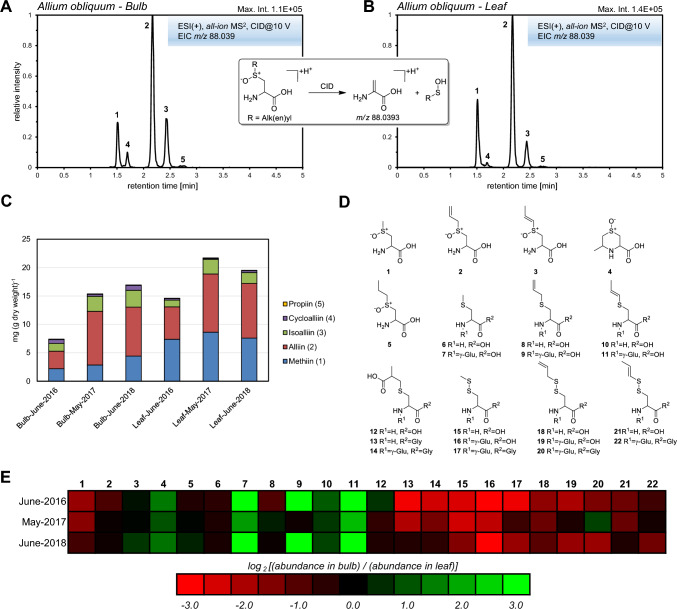
Detection of *S*-alk(en)ylcysteine sulfoxides in hydromethanolic *A. obliquum* bulb (**A**) and leaf (**B**) extracts using all-ion fragmentation. *S*-Alk(en)ylcysteine sulfoxides **1**–**5** were detected via their common fragment ion (protonated dehydroalanine) at *m/z* 88.039. Chromatograms were obtained using UHPLC/ESI-QTOFMS operated in positive ion mode and chromatographic method A at a collision energy of 10 V. CID mass spectra of **1**–**5** are given in Supplemental Fig. 7. Quantification of alk(en)ylcysteine sulfoxides in bulb and leaf tissue of *Allium obliquum* (C). Molecular structures of annotated cysteine conjugates accumulating in leaves and bulbes of *A. obliquum* (**D**). CID mass spectra of **9**, **12** and **20** are given in Supplemental Fig. 8. Analytical data of **1**–**22** are listed in Supplemental Table 4a. Heatmap representation of log_2_-transformed abundance ratios of **1**–**22** in bulbs and leaves of *A. obliquum* (**E**)

Protonated ACSO form under CID conditions a common fragment ion at *m/z* 88.039 by elimination of alk(en)yl sulfenic acid (Böttcher et al., 2017). To screen for ACSO, leaf and bulb extracts of *A. obliquum* were analyzed in all-ion fragmentation mode using chromatographic method A. Five peaks were registered in both EICs at *m/z* 88.039 (Fig. 3A, B). By comparison with corresponding EICs obtained from bulb extracts of *A. sativum* and *A. cepa* (Supplemental Fig. 6) and by interpretation of CID mass spectra (Supplemental Fig. 7) detected peaks were assigned to methiin (**1**), cycloalliin (**4**), alliin (**2**), isoalliin (**3**) and propiin (**5**). Depending on the cultivation year, the total amount of ACSO in bulbs of *A. obliquum* varied between 0.74 and 1.69% dry weight. (Fig. 3C, Supplemental Table 2). The mass ratio of alliin/methiin/isoalliin/cylcoalliin averaged over three cultivation years was 51/25/18/6. The mass proportion of propiin in total ACSO content was for all three cultivation years below 1%. With alliin as dominating ACSO, *A. obliquum* bulbs displayed a garlic-type ACSO chemotype. However, the relative proportion of methiin and in particular isoalliin/cycloalliin in total ACSO content was significantly higher in *A. obliquum* bulbs in comparison to *A. sativum* bulbs (average mass ratio of alliin/methiin/isoalliin in fresh bulbs of 14 semi-bolting accessions: 87/11/2 (Hornickova et al., 2010)).

At the developmental stage studied, the total amount of ACSO was higher in leaves than in bulbs in each of the cultivation years reaching 1.46–2.17% of dry weight (Fig. 3C). This is in agreement with the observation that the highest levels of ACSO were found in leaves and pseudostems of developing garlic plants (Hornickova et al., 2011) and supports the general assumption that de novo synthesis of ACSO from glutathione occurs primarily in the green parts of *Allium* plants (Yoshimoto & Saito, 2019). Compared to bulbs, the average mass ratio of alliin/methiin/isoalliin/cycloalliin in leaves was shifted in favor of methiin towards 45/43/10/2. The relative proportion of propiin in total ACSO content in leaves was again very low (< 0.5% by mass).

Besides ACSO, a number of biosynthetic intermediates of methiin, alliin and isoalliin were detected in leaf and bulb extracts of *A. obliquum* including deoxymethiin (**6**), deoxyalliin (**8**), deoxyisoalliin (**10**) and corresponding γ-glutamyl conjugates (**7**, **9**, **11**) (Fig. 3D). Due to the low propiin concentration in leaf and bulb tissue, deoxypropiin and γ-glutamyldeoxypropiin were not detected. Likewise, γ-glutamyl conjugates of ACSO, which, for example, accumulate in bulbs of *A. cepa* (Böttcher et al., 2017), were virtually absent. As putative early intermediates in the biosynthesis of alliin, isoalliin and propiin, *S*-(2-carboxypropyl)-substituted cysteine (**12**), cysteinyl-glycine (**13**) and glutathione (**14**) were detectable (Yoshimoto & Saito, 2019). In addition, *S*-(alk(en)ylthio)-substituted cysteines (**15**, **18**, **21**), γ-glutamylcysteines (**16**, **19**) and glutathiones (**17**, **20**, **22**) were identified. These mixed disulfides represent reaction products of alk(en)ylsulfenic acids or dialk(en)ylthiosulfinates with corresponding thiols (Rabinkov et al., 2000). It is currently unclear whether these compounds are formed in vivo during catabolic turnover of ACSO or in vitro during sample preparation, where alliinase-mediated degradation of ACSO cannot be completely blocked. Structures of cysteine conjugates **6**–**22** were established based on CID mass spectra obtained from protonated molecular ions (Supplemental Table 4a). In case of peptide conjugates, characteristic Y, B and Z fragment ions were observed in the CID mass spectra (Supplemental Fig. 8). In addition, indicative fragment ions related to the cysteine side chain (*m/z* 73.011 (C_3_H_5_S^+^), *m/z* 104.983 (C_3_H_5_S_2_^+^), *m/z* 119.016 (C_4_H_7_O_2_S^+^)) were formed upon CID of protonated *S*-propenyl, *S*-propenylthio and *S*-(2-carboxypropyl) cysteine conjugates. Discrimination of isomeric cysteine conjugates with prop-1-enyl or prop-2-enyl side chain was facilitated by comparison with analytical data obtained from corresponding onion and garlic bulb extracts. According to their ACSO chemotype, these extracts mainly contained either prop-1-enyl or prop-2-enyl substituted cysteine conjugates.

To compare metabolite levels in bulb and leaf tissue, peak areas of quantifier ions of cysteine conjugates **6**–**22** were determined and abundance ratios calculated (Fig. 3E, Supplemental Table 3). Strikingly, levels of γ-glutamyl-*S*-alk(en)ylcysteines (**7**, **9**, **11**) were significantly higher in bulb tissue compared to leaf tissue whereas the opposite was observed for most of the *S*-(2-carboxypropyl) (**12**–**14**) and *S*-(alk(en)ylthio)-substituted cysteine derivatives (**15**–**22**). According to the currently accepted biosynthetic scheme, γ-glutamyl-*S*-alk(en)ylcysteines are precursors of ACSO and de novo synthesized from glutathione in green parts of *Allium* plants. In *A. sativum*, they are also discussed as storage peptides, which are transported together with ACSO from green leaves into developing bulbs. During sprouting γ-glutamyl-*S*-alk(en)ylcysteines are converted into ACSO and transported to the emerging leaves (Yoshimoto & Saito, 2019). The observed accumulation of γ-glutamyl-*S*-alk(en)ylcysteines **7**, **9** and **11** in developing *A. obliquum* bulbs may also be related to a function as storage peptide. However, detailed quantitative analyses of bulb and leaf material sampled at different developmental stages are required to substantiate this hypothesis.

### Flavonoids

To screen for aglycones of *O*-glycosylated flavonoid conjugates, bulb and leaf extracts of *A. obliquum* were analyzed at different collision energies in all-ion fragmentation mode using chromatographic method B and the positive ion mode. Based on elemental composition, six putative flavonoid aglycones (C_15_H_10_O_5_, C_15_H_10_O_6,_ C_15_H_10_O_7_, C_16_H_12_O_6_, C_16_H_12_O_7_, C_17_H_14_O_7_) were exclusively detected in leaf extracts, but not in bulb extracts. To elucidate their molecular structures, CID mass spectra were acquired in pseudo-MS^3^ experiments and compared with corresponding spectra from libraries (MassBank, METLIN) and reference compounds. Eight different flavonoid aglycones were annotated including the flavones apigenin, luteolin, chrysoeriol, selgin (5′-hydroxychrysoeriol), tricin and the flavonols kaempferol, quercetin and isorhamnetin (Supplemental Fig. 9). Since no library spectrum or reference compound was available, the annotation of selgin is based on the similarity of its CID mass spectrum to that of chrysoeriol and is therefore only putative. However, as biosynthetic precursor of tricin (Lam et al., 2015), co-occurence of selgin and tricin is plausible from a biochemical viewpoint.

**Fig. 4:**
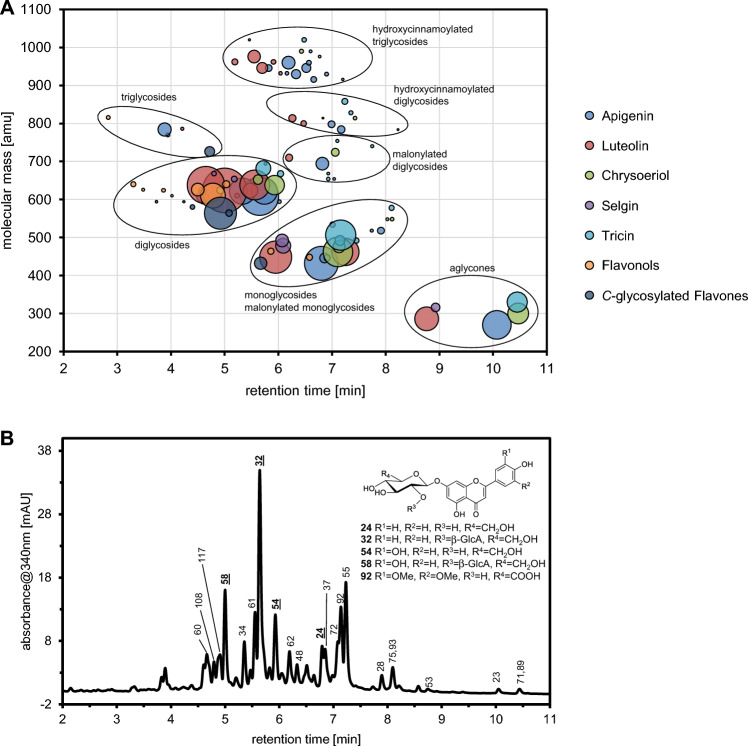
Mass-retention time map of all annotated flavonoids (**23**–**122**) accumulating in *A. obliquum* leaves (**A**). The bubble area is propotional to the average peak area of the quantifier ion of the corresponding flavonoid. Average peak areas were obtained from hydromethanolic leaf extracts (n = 3, cultivation year 2016, 2017, 2018) using UHPLC/DAD/ESI-QTOFMS operated in negative ion mode and chromatographic method B. Extracted wavelength chromatogram (340 nm, bandwidth 4 nm) obtained from a hydromethanolic *A. obliquum* leaf extract (**B**). Structures of the identified flavone glycosides are shown. For compound labelling see Supplemental Table 5b–h

For structural characterization of the *O*-glycosylated flavonoids, precursor ions of the aglycone fragment ions were identified from all-ion fragmentation data using low collision energy scans. Afterwards, CID mass spectra of the identified [M + H]^+^ ions were recorded in targeted-MS^2^ mode. Since glycosylated flavonoids are also very well electrospray ionized in negative ion mode, CID mass spectra of the corresponding [M − H]^−^ ions were additionally acquired. By combined interpretation of the obtained CID mass spectra (Kachlicki et al., 2016), a total of 29 apigenin (**24**–**52**, Supplemental Table 4b), 17 luteolin (**54**–**70**, Supplemental Table 4c), 11 chrysoeriol (**72**–**82**, Supplemental Table 4d), five selgin (**84**–**88**, Supplemental Table 4e),16 tricin (**90**–**105**, Supplemental Table 4f), four kaempferol (**106**–**109**, Supplemental Table 4 g), five quercetin (**110**–**114**, Supplemental Table 4 g) and one isorhamnetin conjugate (**115**, Supplemental Table 4 g) were annotated in leaf extracts. In addition, apigenin (**23**), luteolin (**53**), chrysoeriol (**71**), selgin (**83**) and tricin (**89**) were also detectable in unconjugated form. A corresponding mass-retention time map of all annotated flavonoids is given in Fig. 4A.

The 78 annotated flavone *O*-glycosides comprise 19 mono-, 37 di- and 22 triglycosides with a maximum of one *O*-acyl moiety linked to the glycone. As glycone building blocks, hexoses and hexuronic acids were exclusively detected. Five of the flavone mono-*O*-glycosides and six of the flavone di-*O*-glycosides contain an *O*-malonyl-hexosyl moiety. Differently substituted *O*-hydroxycinnamoyl-hexuronosyl moieties are present in nine of the flavone di-*O*-glycosides and 19 of the flavone tri-*O*-glycosides. The most frequently detected hydroxycinnamoyl moieties were feruloyl (in 16 conjugates) and coumaroyl (in 8 conjugates). In contrast, caffeoyl and sinapoyl moieties were only found in four and one flavone glycoconjugate, respectively. It should be noted, that CID mass spectra of protonated and deprotonated hydroxycinnamoylated flavone di-*O*- and tri-*O*-glycosides display informative Y and B ions, which allow complete analysis of the glycone sequence. Fully interpreted CID mass spectra of **39** (apigenin ← Hex ← (Feruloyl-HexA)) and **103** (tricin ← HexA ← (Coum-HexA) ← Hex) are exemplarily shown in Supplemental Fig. 10 and 11, respectively.

The diversity of the detected flavone *O*-glycosides did not only result from the structural diversity of aglycone and glycone building blocks, but also from different glycosylation sites on the aglycone. For example, two apigenin *O*-hexuronides (**25**, **26**) and three apigenin di-*O*-hexuronides (**33**, **34**, **35**), two of which carry diglycosidic glycones (**34**, **35**), were detetcted. The number and type of these isomers may indicate that apigenin is *O*-hexuronylated at two different positions in leaves of *A. obliquum*.

To gain a more detailed insight into the molecular structures of foliar *A. obliquum* flavonoids, some of the quantitatively dominating flavone glycosides were isolated by semi-preparative HPLC. Using NMR spectroscopy and sugar analysis (Fig. 4B) **32** was identified as apigenin 7-*O*-β-(2´´-*O*-β-glucuronopyranosyl)-glucopyranoside, **58** as luteolin 7-*O*-β-(2´´-*O*-β-glucuronopyranosyl)-glucopyranoside and **92** as tricin 7-*O*-β-glucuronopyranoside. In addition, apigenin 7-*O*-β-glucopyranoside (**24**) and luteolin 7-*O*-β-glucopyranoside (**54**) were identified using authentic reference compounds.

In comparison to flavone *O*-glycosides, the number and the structural diversity of the detected flavonol *O*-glycosides were significantly lower. In total, only two flavonol mono-*O*-glycosides, seven di-*O*-glycosides and one tri-*O*-glycoside with hexosyl and hexuronosyl moieties were annotated. Using reference compounds, two flavonol mono-*O*-glycosides were identified as kaempferol 3-*O*-β-glucopyranoside (**106**) and quercetin 3-*O*-β-glucopyranoside (**110**). Acylated flavonol *O*-glycosides could not be detected. Interpreted CID mass spectra of the quantitatively dominating kaempferol di-*O*-hexoside **108** are given in Supplemental Fig. 12.

Beside *O*-glycosylated flavones and flavonols also *C*-glycoslyated flavones are produced in leaves of *A. obliquum*. Based on the characteristic ion series formed upon CID of the deprotonated molecular ions by cross-ring cleavage of the C-glycosyl moieties, six *C*-glycosylated apigenin (**116**–**121**) and one *C*-glycosylated luteolin conjugate (**122**) were annotated (Supplemental Table 5 h). Hexose and pentose were identified as *C*-glycosidically bound sugars. In the apigenin conjugates **119** and **121** both *C*-glycosyl and *O*-glycosyl moieties were detected. Among the *C*-glycoslyated flavones, apigenin *C*-hexoside *C*-pentoside (**117**) was the most abundant one. Interpreted CID mass spectra of **117** and **121** are given in Supplemental Fig. 13.

**Fig. 5:**
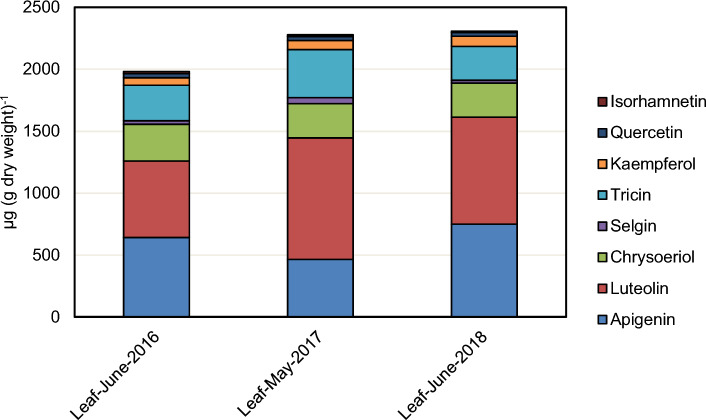
Quantification of flavonoid aglycones in leaves of *A. obliquum*. Hydromethanolic leaf extracts were enzymatically hydrolysed and released flavonoid aglycones quantified by UHPLC/ESI-QTOFMS

In order to quantify the level of flavonoid aglycones in leaf tissue, acidic and enzymatic hydrolysis procedures were tested and optimized. Incubation of a hydromethanolic leaf extract with a mixture of β-glucosidase and β-glucuronidase for 4 h at pH 5.0 and 40 °C led to hydrolysis of most of the *O*-glycosylated flavonoids. However, hydroxycinnamoylated flavone *O*-glycosides resisted enzymatic hydrolysis even after prolonged incubation (22 h, 40 °C). Compared to enzymatic hydrolysis, acidic hydrolysis of hydromethanolic leaf extracts (1 M HCl, 80 °C, 90 min) resulted in significantly lower yields of flavone aglycones which could not be increased by either longer reaction times or higher reaction temperatures. Therefore, enzymatic hydrolysis was applied for quantification of flavonoid aglycones. Since the used enzymes were unable to hydrolyze hydroxycinnamoylated flavone *O*-glycosides and flavone *C*-glycosides the reported flavone levels are systematically underestimated. The total content of flavonoid aglycones in leaves of *A. obliquum* after enzymatic hydrolysis ranged from 0.20 to 0.23% of dry weight (Fig. 5, Supplemental Table 2). In hydrolyzed bulb extracts, levels of flavonoid aglycones were below the detection limit. Flavones accounted for 94–95% by mass of the total flavonoid aglycone content in leaves with the remainder being flavonols. Among the flavones, apigenin (465–751 µg/g dry weight) and luteolin (617–981 µg/g dry weight) were quantitatively dominating followed by tricin (274–389 µg/g dry weight) and chrysoeriol (276–297 µg/g dry weight). Kaempferol (61–81 µg/g dry weight) was the most abundant flavonol.

### Steroidal saponins

Steroidal saponins and sapogenins have been identified so far in over 40 different *Allium* species (Sobolewska et al., 2016). To screen for steroidal saponins in *A. obliquum*, all-ion fragmentation data obtained from bulb and leaf extracts using chromatographic method B and the positive ion mode were analyzed. At a collision energy of 30 V, three putative protonated sapogenins with elemental compositions of C_27_H_43_O_4_^+^, (*m/z* 431.3156), C_27_H_43_O_3_^+^ (*m/z* 415.3207), C_27_H_45_O_3_^+^ (*m/z* 417.3363) were detected in bulb extracts and in traces also in leaf extracts (Supplemental Fig. 14). Inspection of low collision energy scans revealed the presence of 10 putative steroidal saponins (**123**–**132**, Table 1). Saponins **123**–**131** were detected within a relatively narrow retention time range of 7.4–8.7 min. In contrast, saponin **132** eluted at 14.1 min. Under the negative ion electrospray conditions applied, the saponins **123**–**132** formed [M − H]^−^ and [M + HCOO]^−^ ions. Under positive ion electrospray conditions, formation of [M + Na]^+^, [M + NH_4_]^+^ and numerous in-source fragment ions, mainly by cleavage of glycosyl moieties, was observed. In addition, the early eluting saponins **123**–**131** formed prominent [M + H − H_2_O]^+^ ions, whereas the late eluting saponin **132** did not. In accordance with the literature (Kite et al., 2007), the observed chromatographic and mass spectral behavior suggest, that **123**–**131** are furostanol saponins with an open glycosylated side chain and a hemiketal at C-22 and **132** a spirostanol saponin with a closed spiroketal ring at C-22. Small amounts of the quantitatively dominating saponin **124** were isolated by semi-preparative HPLC and treated for 24 h with a β-glucosidase. Analysis of the reaction mixture by LC/MS revealed formation of the saponin **132** and a sapogenin with the elemental composition C_27_H_42_O_4_ proving that **124** is a furostanol saponin and **132** its corresponding spirostanol analog.

**Table 1:** Analytical data of steroidal saponins accumulating in bulbs of *A. obliquum*

No	t_R_ [min]	Characteristic ion	Elemental composition		Total glycone composition	Type	Observed fragment ions upon CID^b^
		Measured *m/z*^a^, type	*Saponin*	*Sapogenin*			Precursor ion@collision energy: *m/z* (rel. intensity [%])
**123**	7.46	1049.5143, [M + H − H_2_O]^+^ 1065.5102, [M − H]^−^	C_50_H_82_O_24_	C_27_H_44_O_5_	Hex_3_-Pent	Furostanol	***m/z* 1049@20 V:** 1049 (41), 917 (3), 887 (11), 755 (12), 725 (28), 593 (67), 457 (10), 431 (100), 325 (4), 295 (18), 287 (3), 259 (6)
**124**	7.59	1019.5063, [M + H − H_2_O]^+^ 1035.5015, [M − H]^−^	C_49_H_80_O_23_	C_27_H_44_O_5_	Hex_2_-Pent_2_	Furostanol	***m/z* 1019@20 V:** 1019 (59), 887 (3), 857 (8), 755 (2), 725 (23), 593 (31), 563 (3), 431 (100), 427 (1), 295 (4), 287 (3), 265 (2)
**125**	7.74	887.4632, [M + H − H_2_O]^+^ 903.4594, [M − H]^−^	C_44_H_72_O_19_	C_27_H_44_O_5_	Hex_2_-Pent	Furostanol	***m/z* 887@20 V:** 887 (37), 755 (10), 725 (37), 593 (59), 431 (100), 413 (3), 287 (6), 167 (5)
**126**	8.29	755.4213, [M + H − H_2_O]^+^ 771.4162, [M − H]^−^	C_39_H_64_O_15_	C_27_H_44_O_5_	Hex_2_	Furostanol	***m/z* 755@20 V:** 755 (38), 593 (100), 449 (12), 431 (55), 413 (7), 299 (4), 287 (8), 269 (5), 167 (6)
**127**	8.45	1225.5822, [M + H − H_2_O]^+^ 1241.5784, [M − H]^−^	C_57_H_94_O_29_	C_27_H_44_O_4_	Hex_5_	Furostanol	***m/z* 1225@30 V:** 1225 (23), 1063 (3), 901 (4), 739 (48), 577 (100), 487 (9), 433 (9), 415 (49), 397 (14), 325 (48), 271 (19), 253 (9), 163 (18)
**128**	8.57	1063.5304, [M + H − H_2_O]^+^ 1079.5270, [M − H]^−^	C_51_H_84_O_24_	C_27_H_44_O_4_	Hex_4_	Furostanol	***m/z* 1063@30 V:** 1063 (60), 739 (13), 577 (46), 559 (9), 487 (2), 433 (3), 415 (100), 397 (33), 379 (5), 325 (29), 271 (37), 253 (24), 167 (10), 163 (42)
**129**	8.60	1003.5089, [M + H − H_2_O]^+^ 1019.5051, [M − H]^−^	C_49_H_80_O_22_	C_27_H_44_O_4_	Hex_2_-Pent_2_	Furostanol	***m/z* 1003@30 V:** 1003 (100), 871 (2), 841 (39), 739 (5), 709 (12), 577 (18), 559 (7), 433 (2), 427 (2), 415 (62), 397 (46), 295 (10), 283 (6), 271 (49), 265 (12), 259 (6), 253 (51), 229 (9), 211 (10), 167 (12)
**130**	8.55	1227.5979, [M + H − H_2_O]^+^ 1243.5938, [M − H]^−^	C_57_H_96_O_29_	C_27_H_46_O_4_	Hex_5_	Furostanol	***m/z* 1227@30 V:** 1227 (61), 1065 (4), 903 (4), 741 (49), 579 (100), 487 (8), 435 (21), 417 (63), 399 (6), 325 (62), 289 (5), 273 (35), 255 (4), 167 (4), 163 (38)
**131**	8.68	1065.5457, [M + H − H_2_O]^+^ 1081.5413, [M − H]^−^	C_51_H_86_O_24_	C_27_H_46_O_4_	Hex_4_	Furostanol	***m/z* 1065@30 V:** 1065 (100), 903 (7), 741 (10), 579 (36), 487 (2), 435 (10), 417 (70), 399 (13), 325 (28), 273 (49), 255 (9), 167 (8), 163 (30)
**132**	14.09	874.4803, [M + NH_4_]^+^ 855.4370, [M − H]^−^	C_43_H_68_O_17_	C_27_H_42_O_4_	Hex-Pent_2_	Spirostanol	***m/z* 874@40 V**: 431 (100), 413 (57), 395 (10), 295 (2), 287 (12), 265 (5), 259 (5), 247 (8), 229 (9), 211 (11)

Fully annotated accurate-mass CID mass spectra are given in Supplemental Table 4i. *m/z* of the fragment ions [aglycone + H − H_2_O]^+^ (for **123**–**131**) and [aglycone + H]^+^ (for **132**) are underlined

^a^Mass accuracy ranged from −0.9 to 2.2 ppm

^b^Fragment ions with *m/z* < 150 are not shown

Upon CID, [M + H − H_2_O]^+^ ions of furostanol saponins **123**–**131** formed series of [Y_i_ − H_2_O]^+^ ions and a prominent [Y_0_ − H_2_O]^+^ ion by sequential losses of glycosyl moieties (Supplemental Table 4i). In addition, in some cases also informative B ions were formed. For instance, in the CID mass spectra obtained from [M + H − H_2_O]^+^ of the major furostanol saponin **124**, low abundant B_3_^+^ ions at *m/z* 427.143 (C_16_H_27_O_13_^+^, [Hex-Pent_2_ + H]^+^) and B_2_^+^ at *m/z* 295.102 (C_11_H_19_O_9_^+^, [Hex-Pent + H]^+^) were registered (Supplemental Fig. 15). Both ions were also observable in the CID mass spectrum obtained from [M + NH_4_]^+^ of the corresponding spirostanol saponin **132** (Supplemental Fig. 16). This suggests that **124** is a bisdesmoside carrying a hexosyl moiety at C-26-OH in the side chain and a trisaccharide moiety (Hex-Pent_2_) at a hydroxyl group of the steroid nucleus. CID mass spectra obtained from [M + H − H_2_O]^+^ of furostanol saponins **123**–**131** display further indicative fragment ions of the type [Y_0_ − H_2_O − C_8_H_16_O_2_]^+^, which result from the characteristic cleavage of the E-ring (Liang et al., 2002).

Negative ion CID mass spectra obtained from [M − H]^−^ or [M + HCOO]^−^ ions of furostanol saponins **123**—**131** also show series of Y ions arising from sequential losses of glycosyl moieties (Supplemental Table 4i). However, even at higher collision energies relative intensities of Y_0_^−^ ions are comparatively low (< 10%), and mass spectra are dominated by nonspecific ions with *m/z* < 200 formed by fragmentation of glycosyl moieties.

Furostanol saponins **124** and **125** and spirostanol saponin **132** were the quantitatively dominating saponins in bulb tissue of *A. obliquum* and accounted for approximately 80–90% of the total saponin content. Most of the saponins were also detectable in leaf tissue, but levels reached on average only 1/50 of those in bulb tissue (Supplemental Table 3).

### Hydroxycinnamic acid conjugates

**Fig. 6:**
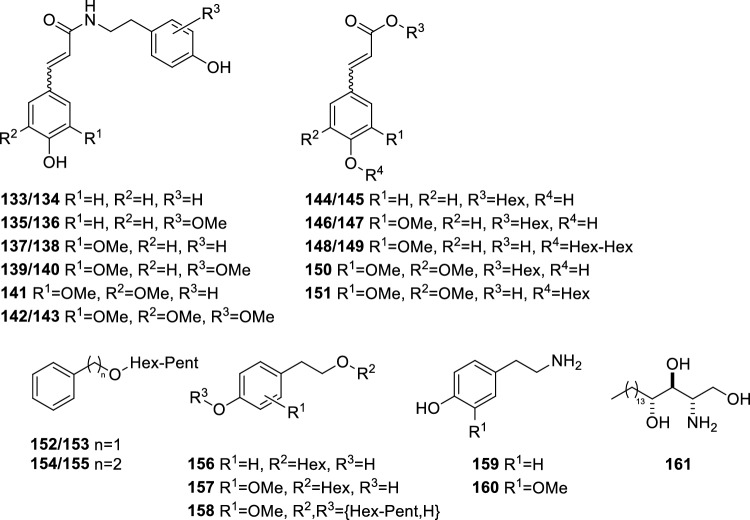
Structures of hydroxycinnamic acid conjugates, phenylethanoids and phytosphingosine accumulating in leaf and bulb tissue of *A. obliquum*

In addition to hydroxycinnamoylated flavone glycosides, a number of other hydroxycinnamic acid conjugates were detected in leaf and bulb extracts, including the hydroxycinnamic acid amides (HCAA) **133**–**143** as well as the glycosylated hydroxycinnamic acids (HCA) **144**–**151** (Fig. 6). HCAA **133**–**143** represent conjugates of coumaric, ferulic or sinapic acid with tyramine or methoxytyramine. Upon CID, [M + H]^+^ ions of **133**–**143** formed characteristic acyl ions at *m/z* 147.044 (coumaroyl, C_9_H_7_O_2_^+^), *m/z* 177.055 (feruloyl, C_10_H_9_O_3_^+^) or *m/z* 207.065 (sinapoyl, C_11_H_11_O_4_^+^) arising from cleavage of the amide bond (Supplemental Fig. 17, Supplemental Table 4j). Complementary fragment ions of the amide bond cleavage consecutively eliminated ammonia and were detected at *m/z* 121.065 (C_8_H_9_O^+^) for tyramine and at *m/z* 151.075 (C_9_H_11_O_2_^+^) for methoxytyramine conjugates. With the exception of sinapoyltyramine (**141**), two chromatographically separated peaks with nearly identical CID mass spectrum were registered for all HCAA, which probably represent pairs of *E*/*Z* isomers. HCAA **133**—**143** were detectable in both leaf and bulb tissue. However, levels of HCAA in leaves exceeded those in bulbs by an average factor of 3.5 (Supplemental Table 3).

The detected glycosylated HCA include two isomeric monohexosides each of coumaric acid (**144**, **145**), ferulic acid (**146**, **147**) and sinapic acid (**150**, **151**) as well as two isomeric dihexosides of ferulic acid (**148**, **149**). CID mass spectra obtained from [M − H]^−^ ions of the monohexosides **144**–**147** and **150** at a collision energy of 10 V showed low abundant ^0,i^X_0_^−^ (i = 2, 3, 4) and [^0,2^X_0_ − H_2_O]^−^ fragment ions by cross-ring cleavage of the hexosyl moiety as well as high abundant Y_0_^−^ and Z_0_^−^ fragment ions (Supplemental Table 4j). In contrast, [M − H]^−^ ions of the monohexoside **151** formed upon CID mainly Y_0_^−^ fragment ions (Supplemental Fig. 18). Likewise, CID mass spectra obtained from [M − H]^−^ ions of the dihexosides **148** and **149** displayed high abundant Y_0_^−^ ions which were accompanied by B_2_^−^ ions suggesting that both isomeric ferulic acid dihexosides carry a diglycosidic glycone. Comparison of the observed fragmentation characteristics with those of isomeric *O*-caffeoyl glucoses and caffeic acid 3-*O*- and 4-*O*-β-glucosides (Jaiswal et al., 2014) suggests, that **144**–**147** and **150** are 1-*O*-hydroxycinnamoylated hexoses, whereas **148**, **149** and **151** are HCA 4-*O*-glycosides. Due to the high pairwise similarity of their CID mass spectra, **144**/**145**, **146**/**147** and **148**/**149** could represent pairs of *E*/*Z* isomers. Most of the glycosylated HCA **144**–**151** were detectable in both leaves and bulbs. Similar as observed for HCAA, leaves accumulated significantly higher levels of **144**–**151** in comparison to bulbs (Supplemental Table 3).

### Other metabolites

Another sixteen metabolites belonging to different biosynthetic classes were putatively annotated (Fig. 6, Supplemental Table 4 k). These include two isomeric benzyl alcohol di-*O*-glycosides (**152**, **153**), *O*-glycosylated phenylethanoids (**154**–**158**), tyramine (**159**), 3-methoxytyramine (**160**) and a series of sphingoid bases (**161**–**167**). CID of [M + NH_4_]^+^ ions of **152** and **153** gave similar fragment ion spectra with B_2_^+^, Y_1_^+^ and Z_0_^+^ fragment ions at *m/z* 295.102 (C_11_H_19_O_9_^+^), *m/z* 271.118 (C_13_H_19_O_6_^+^) and *m/z* 91.054 (C_7_H_7_)^+^ (Supplemental Fig. 19). This suggests, that **152** and **153** are benzyl alcohol *O*-glycosides carrying isomeric pentosyl → hexosyl moieties. The annotated *O*-glycosylated phenylethanoids comprise two isomeric phenylethanol 8-*O*-(*O*-pentosyl-hexosides) (**154**, **155**), a tyrosol 8-*O*-hexoside (**156**), a methoxytyrosol 8-*O*-hexoside (**157**) and a methoxytyrosol *O*-(*O*-pentosyl-hexoside) (**158**). Under ESI conditions, phenylethanoids **154**–**158** formed exclusively [M + NH_4_]^+^ and [M + Na]^+^ ions in the positive ion mode, whereas [M − H]^−^ and [M + HCOO]^−^ were detected in the negative ion mode. Upon CID, [M + NH_4_]^+^ ions of **154**–**158** gave readily interpretable fragment ion spectra with informative B_2_^+^ and Y_1_^+^ fragment ions for **154**, **155** and **158** and Y_0_^+^ and Z_0_^+^ fragment ions for **156**, **157** and **158**. CID mass spectra obtained from [M + HCOO]^−^ of **156** and **157** showed C_1_^−^ at *m/z* 179.056 (C_6_H_11_O_6_^−^) suggesting that in both metabolites the hexosyl moiety is linked with the aglycone via its aliphatic hydroxyl group rather than its aromatic one (Supplemental Fig. 20). The position of the pentosyl → hexosyl moiety in **158** could not be deduced from the acquired CID mass spectra. Metabolites **154**–**160** exhibited similar concentration differences between leaf and bulb tissue as observed for HCA conjugates (Supplemental Table 3). Metabolites **161**–**167** were electrospray-ionized exclusively in the positive ion mode and formed solely [M + H]^+^ ions. The elemental compositions determined for **161**–**167** (C_18_H_35-39_NO_3-4_) pointed to a series of C_18_ sphingoid bases differing in the number of ring and double bond equivalents and in oxygenation (SPB C18:n,O_m_ (n = 0, 1, 2; m = 3, 4)). As a common feature, CID mass spectra obtained from [M + H]^+^ ions of **161**–**167** displayed multiple losses of water and formaldehyde from the precursor ion as well as a characteristic fragment ion at *m/z* 60.044 (C_2_H_6_NO^+^) originating from cleavage of the C2-C3 bond of the sphingoid base (Ryan et al., 2017) (Supplemental Fig. 21, Supplemental Table 4 k). SPB C18:0,O_3_ (**161**) and SPB C18:1,O_3_ (**162**) were the quantiatviely dominating free sphingoid bases in bulb and leaf tissue of *A. obliquum* of which **161** was identified as phytosphingosine using an authentic reference compound.

## Concluding remarks

Due to its unique flavor profile, twisted-leaf garlic represents a promising aroma plant with high potential for domestication and cultivation. To comprehensively explore and structurally characterize polar and semi-polar phytochemicals accumulating in leaves and bulbs of this wild *Allium* species, an UHPLC/ESI-QTOFMS-based workflow originally developed for metabolite profiling of onion bulbs was applied. Using these analytical workflow important metabolite classes influencing nutritional, sensory and technological properties were characterized, including FOS, *S*-substituted cysteine conjugates, flavonoids, steroidal saponins and hydroxycinnamic acid conjugates.

Leaves and bulbs of *A. obliquum* were found to accumulate a complex mixture of FOS including inulin- and *neo*-inulin-type FOS with exclusively β-(2 → 1) Fru*f*-Fru*f* linkages as well as mixed-type FOS with β-(2 → 1) and β-(2 → 6) Fru*f*-Fru*f* linkages. By interpretation of CID mass spectra and by comparison of FOS profiles of onion and garlic with those of twisted-leaf garlic isomeric FOS with a DP of 3–5 were structurally characterized. For further structural analysis of FOS with higher DP and fructans analytical methods of polysaccharide analysis have to be applied. Leaf and bulb material used for analyses of NSC were obtained at the end of the vegetative growth phase. It can be assumed, that the level of FOS/fructans in bulbs of *A. obliquum* and the number-average DP will increase during the further course of the vegetation period similar as observed for garlic (Baumgartner et al., 2000).

Screening experiments of ACSO in leaves and bulbs of *A. obliquum* revealed the presence of alliin, methiin, isoalliin, cycloalliin and traces of propiin. Hence, *A. obliquum* does not form previously unknown or unusual ACSO, such as butiin or homoisoalliin identified in *A. siculum* and *A. tripedale* (Kubec et al., 2010). Alliin is the predominating ACSO in bulbs of *A. obliquum*. In comparison to garlic bulbs, alliin is accompanied by comparatively high relative amounts of methiin and isoalliin, which in combination produce upon processing the unique flavor of *A. obliquum* bulbs. In the developmental stage studied, a great variety of flavonoids, including *O*- and *C*-glycosylated flavones as well as *O*-glycosylated flavonols was exclusively detected in leaves, but not in bulbs of *A. obliquum*. This stands in striking contrast to the massive flavonoid accumulation in bulbs of *A. cepa* cultivars with colored scales (Slimestad et al., 2007). However, it remains to be investigated whether the flavonoid levels of bulbs increase or remain below the detection limit during the further course of the vegetation period until maturity. Compared to flavonoids, an almost inverse distribution pattern between leaf and bulb tissue was observed for the detected steroidal saponins. Using accurate-mass tandem mass spectrometric techniques, only type (furo-, spirostanol) and elemental composition of the aglycone as well as glycan composition could be determined. To gain more detailed insights into molecular structure of these saponins, isolation of quantitatively dominating representatives such as **124**, **125** and **132** and NMR spectroscopic analyses are required in future studies.

### Supplementary Information

Below is the link to the electronic supplementary material.

Supplementary file1 (PDF 2553 kb)

Supplementary file2 (XLSX 88 kb)

## Data Availability

No data have been deposited in public databases for this manuscript.

## Declarations

### Research involving in human and animal participants

This article does not contain any studies with human participants or animals performed by any of the authors.
