# Supplementary material for: Characterization of phytochemicals from twisted-leaf garlic (Allium obliquum L.) using liquid chromatography coupled with electrospray ionization quadrupole time-of-flight mass spectrometry
Source: Metabolomics. 2023 Oct 21;19(11):89. doi: 10.1007/s11306-023-02054-2 (PMC10590303; doi:10.1007/s11306-023-02054-2)
Supplement: Supplementary file 1 — Supplementary file1 (PDF 2553 kb) [file 11306_2023_2054_MOESM1_ESM.pdf]

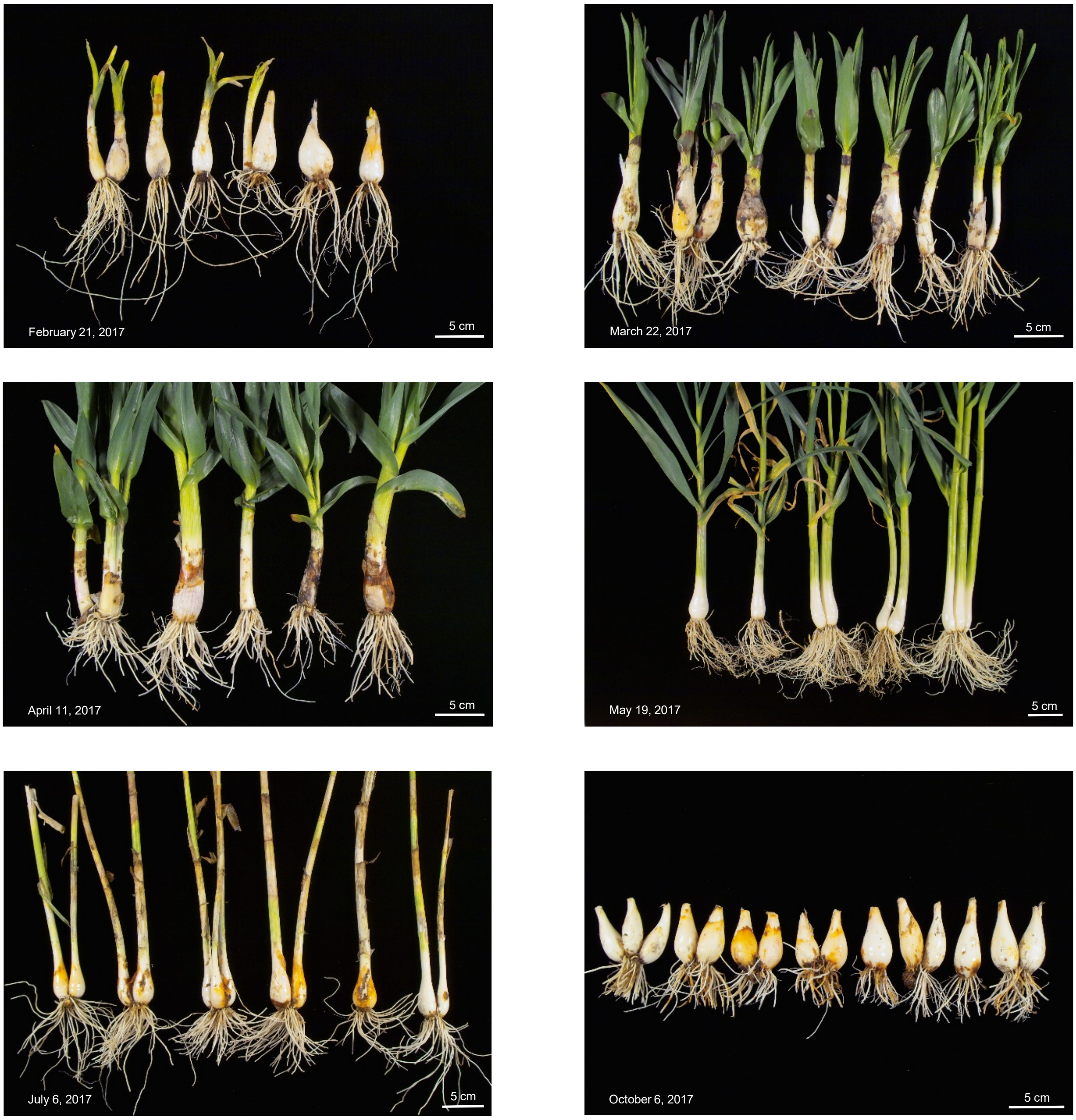

Supplemental Fig. 1 Developmental stages of *A. obliquum* during the growing season 2017. Plants harvested on May 19 were used for metabolite analysis. Roots were shortened due to space limitations. Senescent aerial parts were removed from bulbs harvested on October 6.

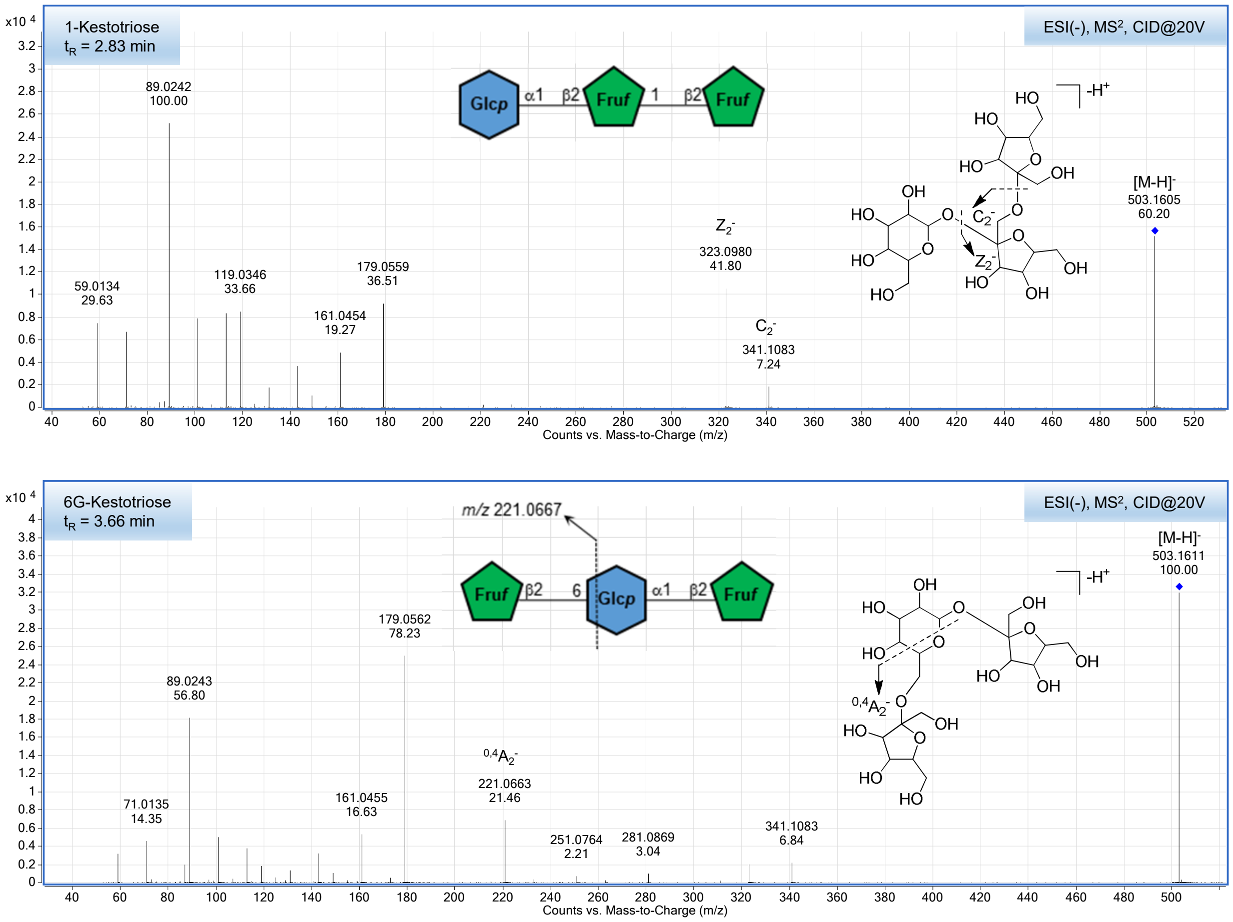

Supplemental Fig. 2 CID mass spectra of kestotrioses detected in a hydromethanolic bulb extract of *A. obliquum*. Spectra were obtained using UHPLC/ESI-QTOFMS and chromatographic method A. Precursor ions are marked with a blue diamond. Identity of 1-kestotriose (1-kestose) was verified using a reference compound.

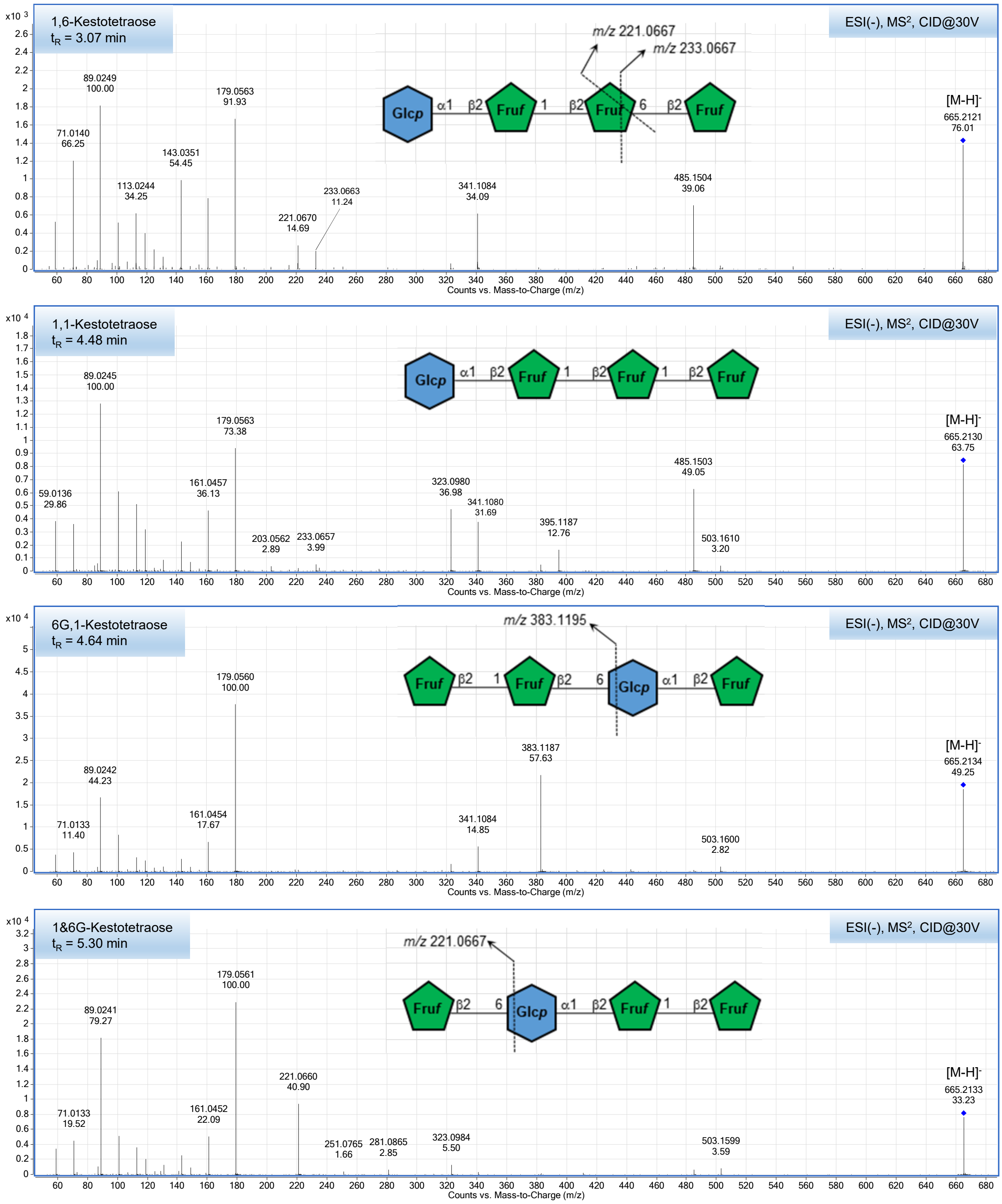

Supplemental Fig. 3 CID mass spectra of kestotetraoses detected in a hydromethanolic bulb extracts of *A. obliquum*. Spectra were obtained using UHPLC/ESI-QTOFMS and chromatographic method A. Precursor ions are marked with a blue diamond. Identity of 1,1-kestotetraose (nystose) was verified using a reference compound.

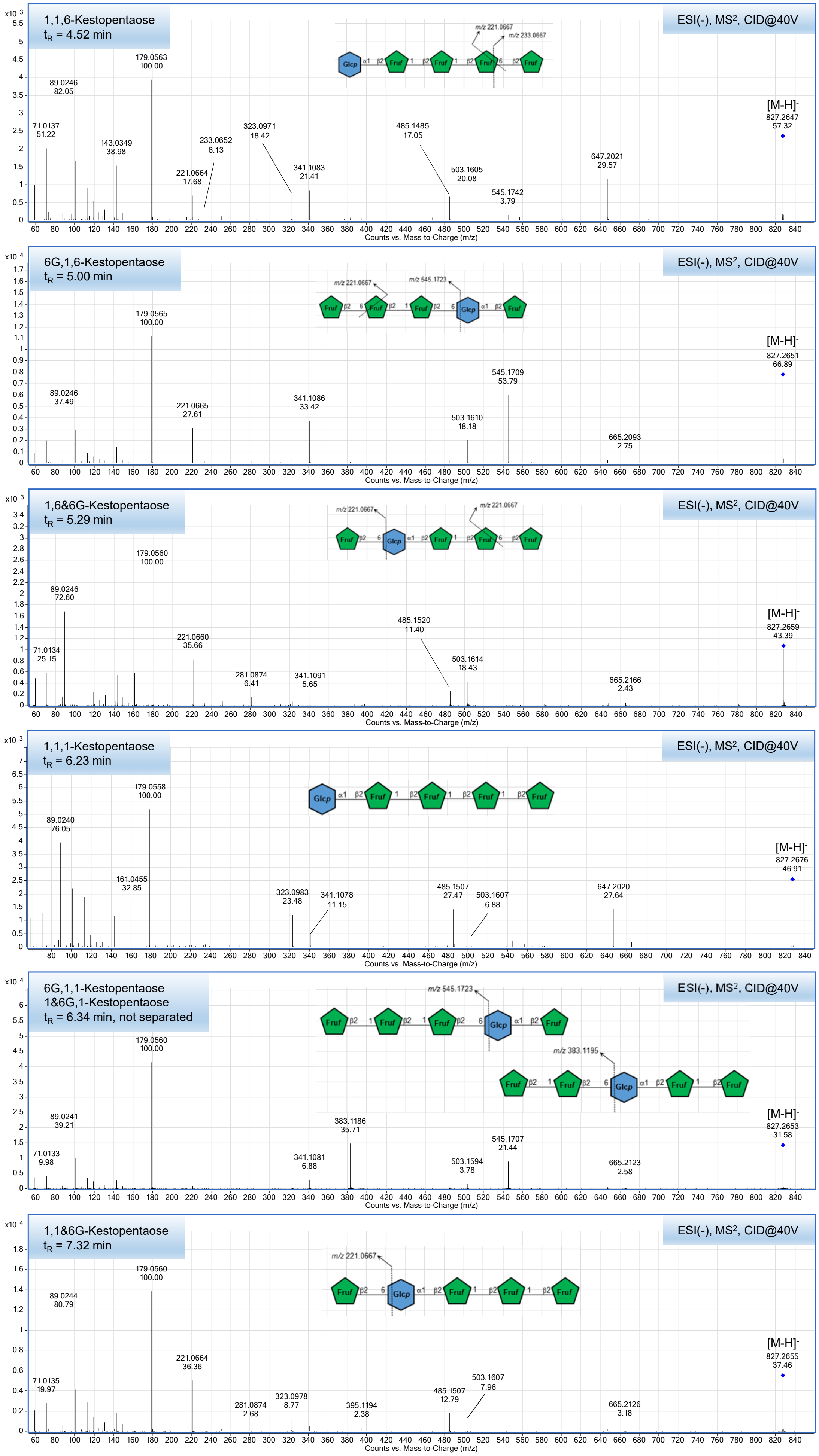

Supplemental Fig. 4 CID mass spectra of kestopentaoses detected in a hydromethanolic bulb extracts of *A. obliquum*. Spectra were obtained using UHPLC/ESI-QTOFMS and chromatographic method A. Precursor ions are marked with a blue diamond.

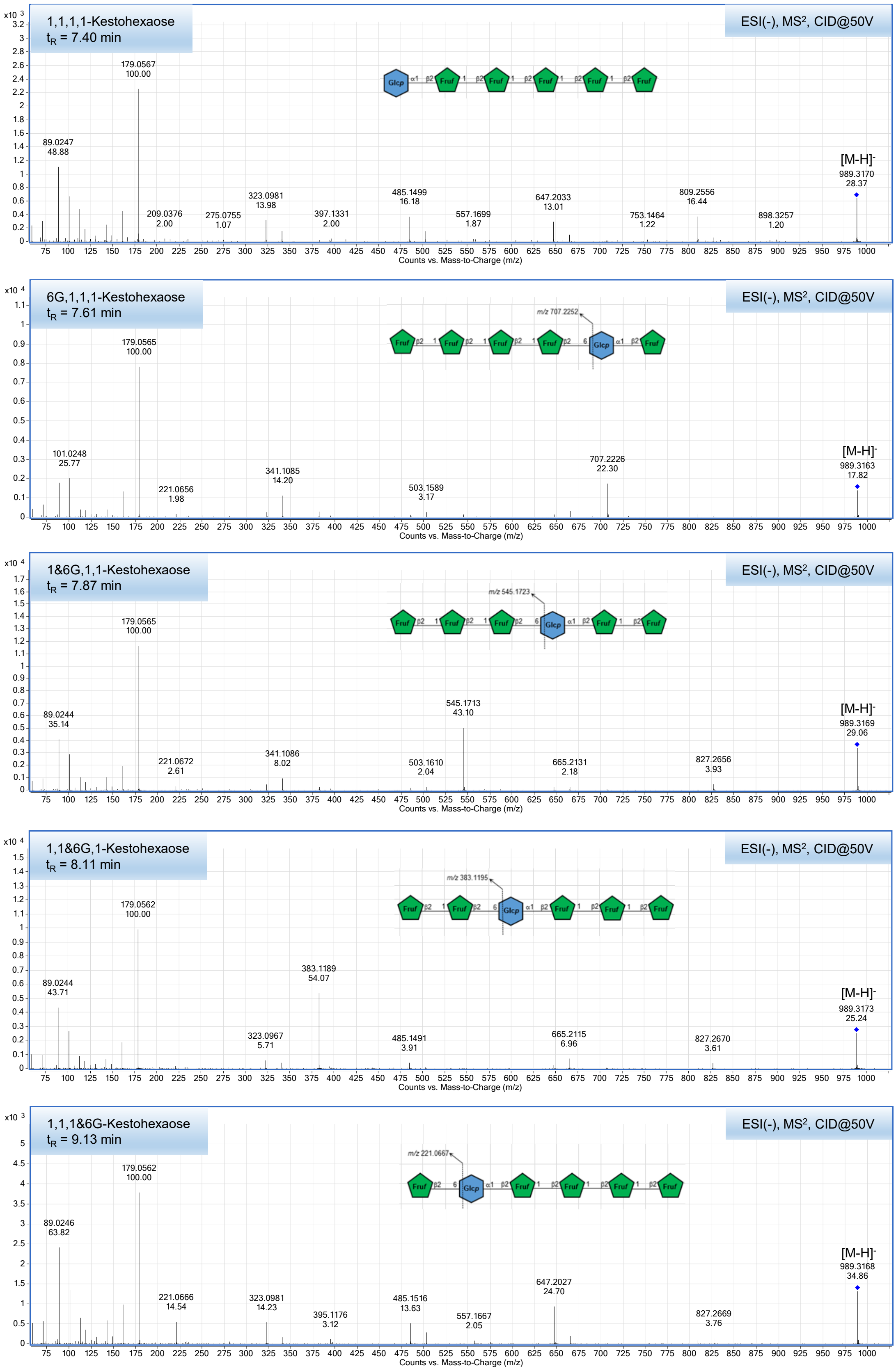

Supplemental Fig. 5 CID mass spectra of annotated inulin and *neo*-inuline-type kestohexaoses detected in a hydromethanolic bulb extract of *A. obliquum*. Spectra were obtained using UHPLC/ESI-QTOFMS and chromatographic method A. Precursor ions are marked with a blue diamond.

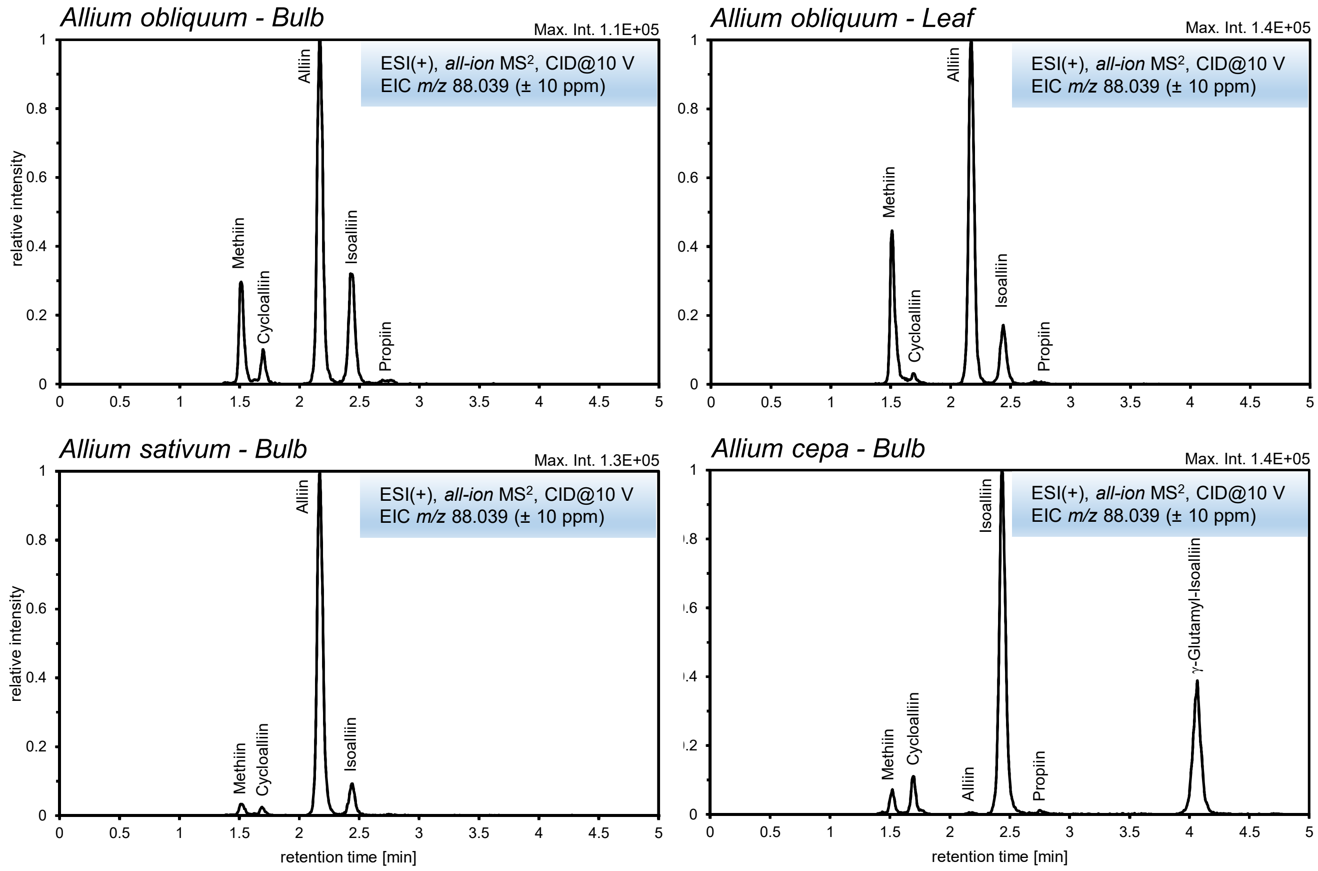

Supplemental Fig. 6 Detection of alk(en)ylcysteine sulfoxides in hydromethanolic bulb extracts of *A. obliquum, A. sativum* and *A. cepa* and a leaf extract of *A. obliquum* using all-ion fragmentation. Alk(en)ylcysteine sulfoxides were detected *via* their common fragment ion (protonated dehydroalanine) at *m/z* 88.039. Chromatograms were obtained using UHPLC/ESI-QTOFMS and chromatographic method A in positive ion mode at a collision energy of 10 V.

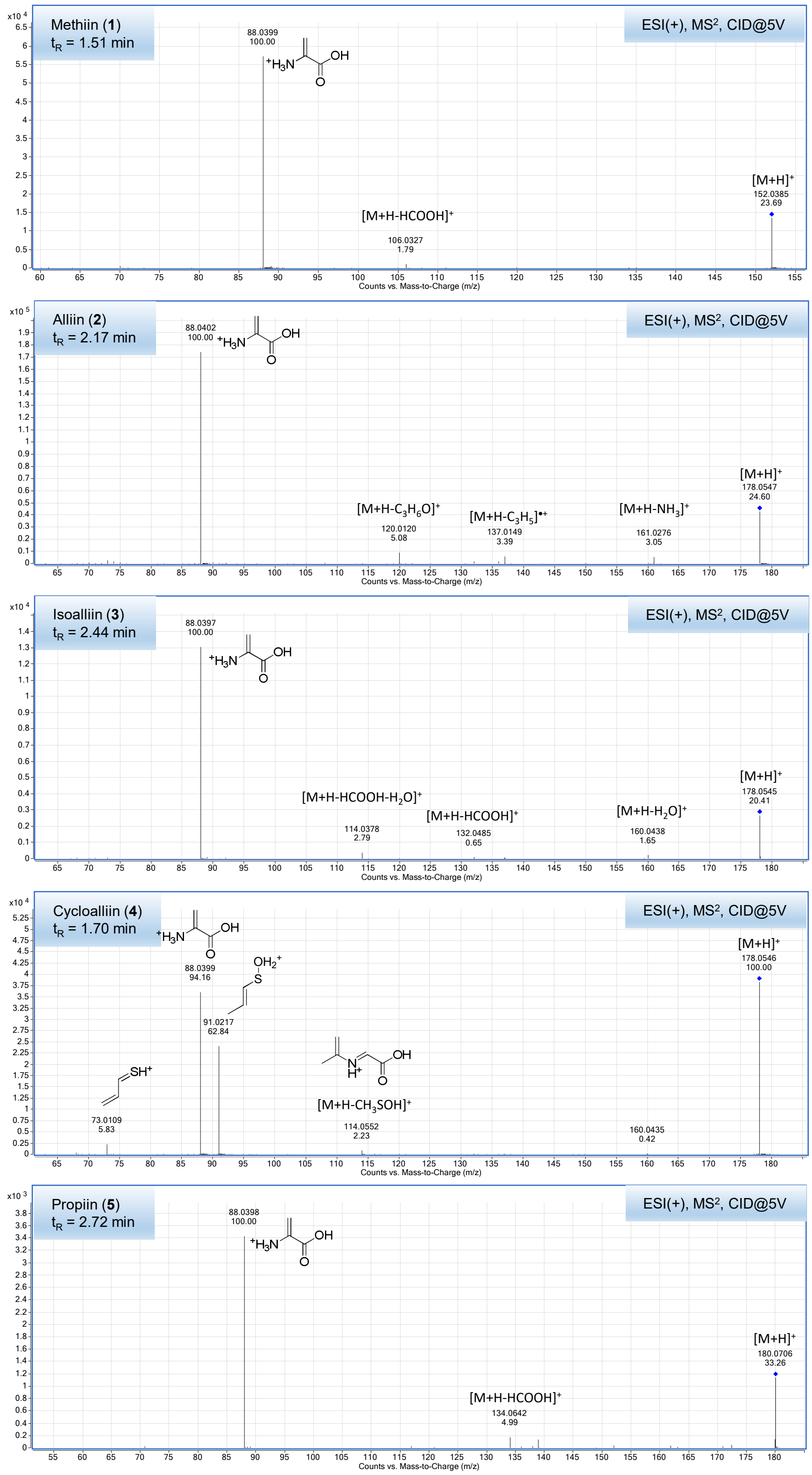

Supplemental Figure 7. CID mass spectra of alk(en)ylcysteine sulfoxides detected in a hydromethanolic bulb extract of *A. obliquum*. Spectra were obtained using UHPLC/ESI-QTOFMS and chromatographic method A. Precursor ions are marked with a blue diamond.

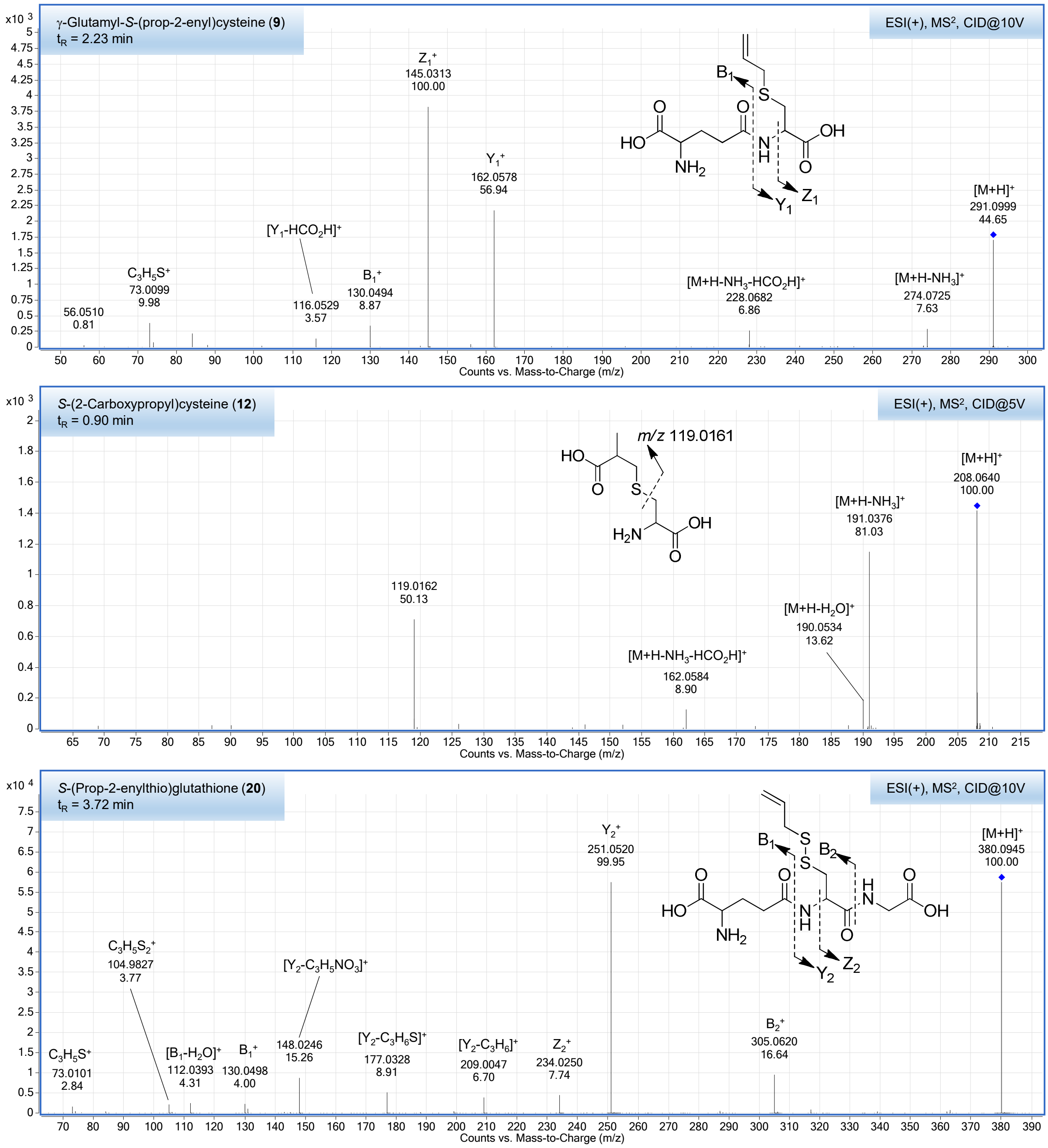

Supplemental Fig. 8 CID mass spectra of cysteine conjugates **9**, **12** and **20** detected in hydromethanolic leaf and bulb extracts of *A. obliquum*. Spectra were obtained using UHPLC/ESI-QTOFMS and chromatographic method B. Precursor ions are marked with a blue diamond.

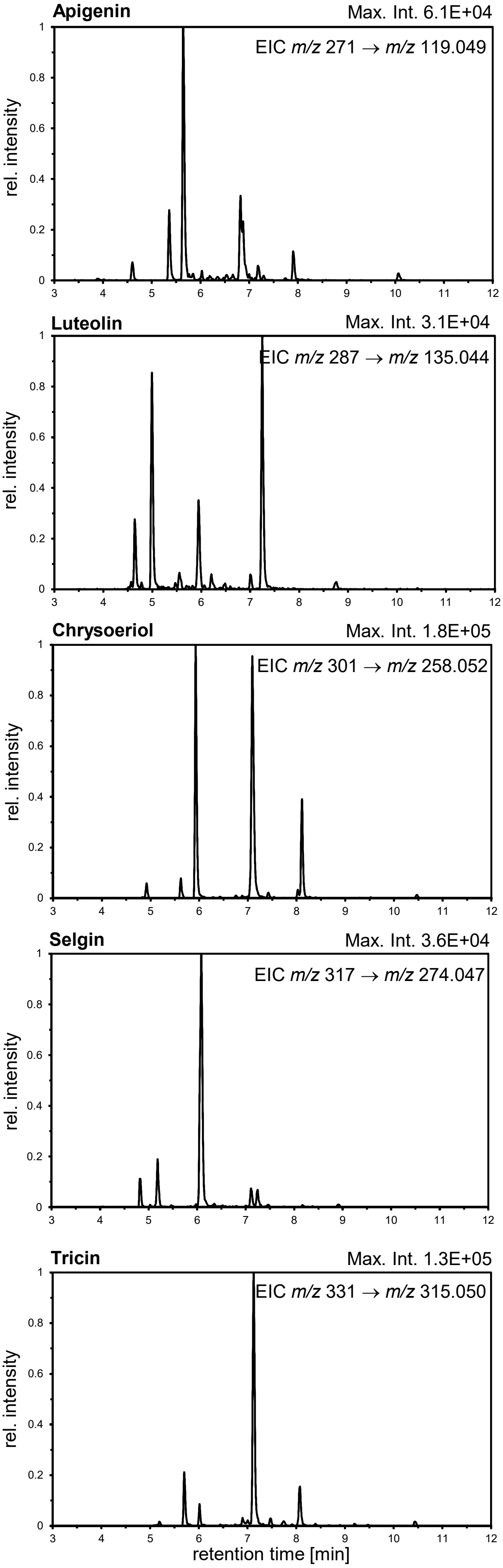

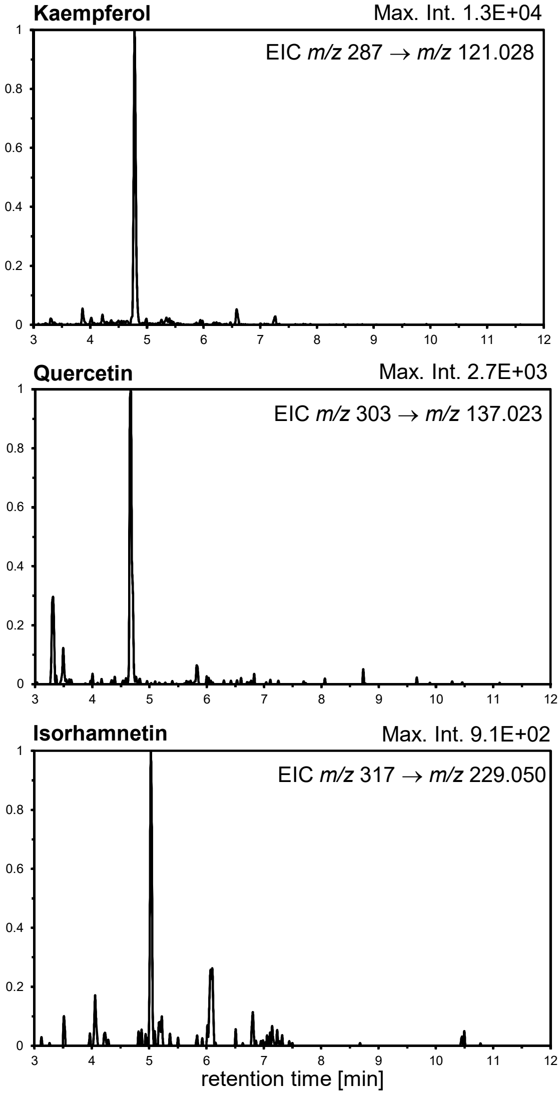

Supplemental Fig. 9 Detection of *O*-glycoslyated flavone and flavonol conjugates in a hydromethanolic *A. obliquum* leaf extract using pseudo-MS^3. Protonated aglycones were generated by in-source fragmentation, selected by Q_1 and fragmented inside the collision cell. Extracted ion chromatograms (EICs) were generated for specific products ions from the obtained product ion spectra.

Chromatograms were obtained using UHPLC/ESI-QTOFMS and chromatographic method B in positive ion mode at a funnel exit voltage of 120 V and a collision energy of 40 V.

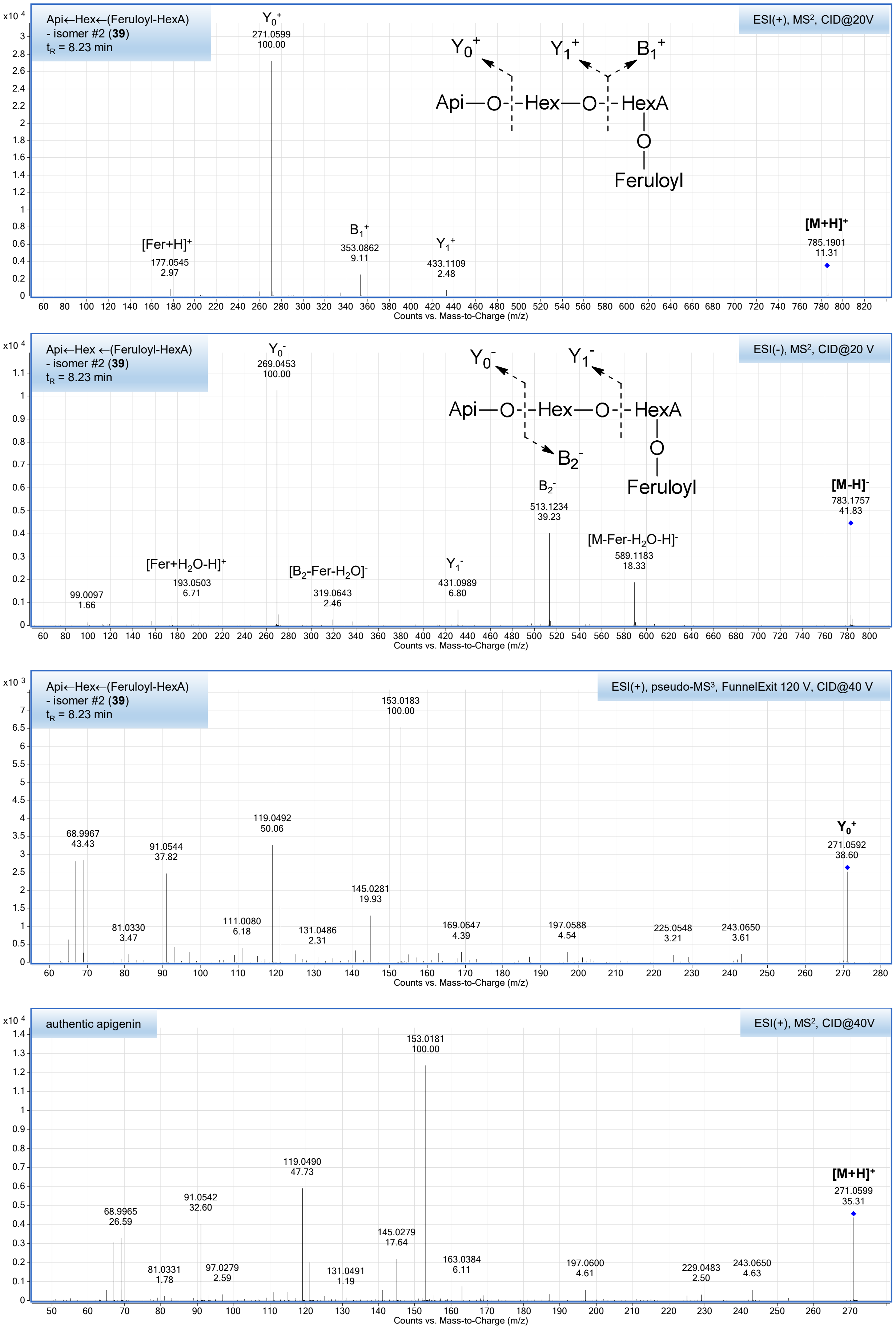

Supplemental Fig. 10 CID mass spectra of the feruloylated apigenin diglycoside **39** detected in a hydromethanolic leaf extract of *A. obliquum* and of authentic apigenin. Spectra were obtained using UHPLC/ESI-QTOFMS and chromatographic method B. Precursor ions are marked with a blue diamond.

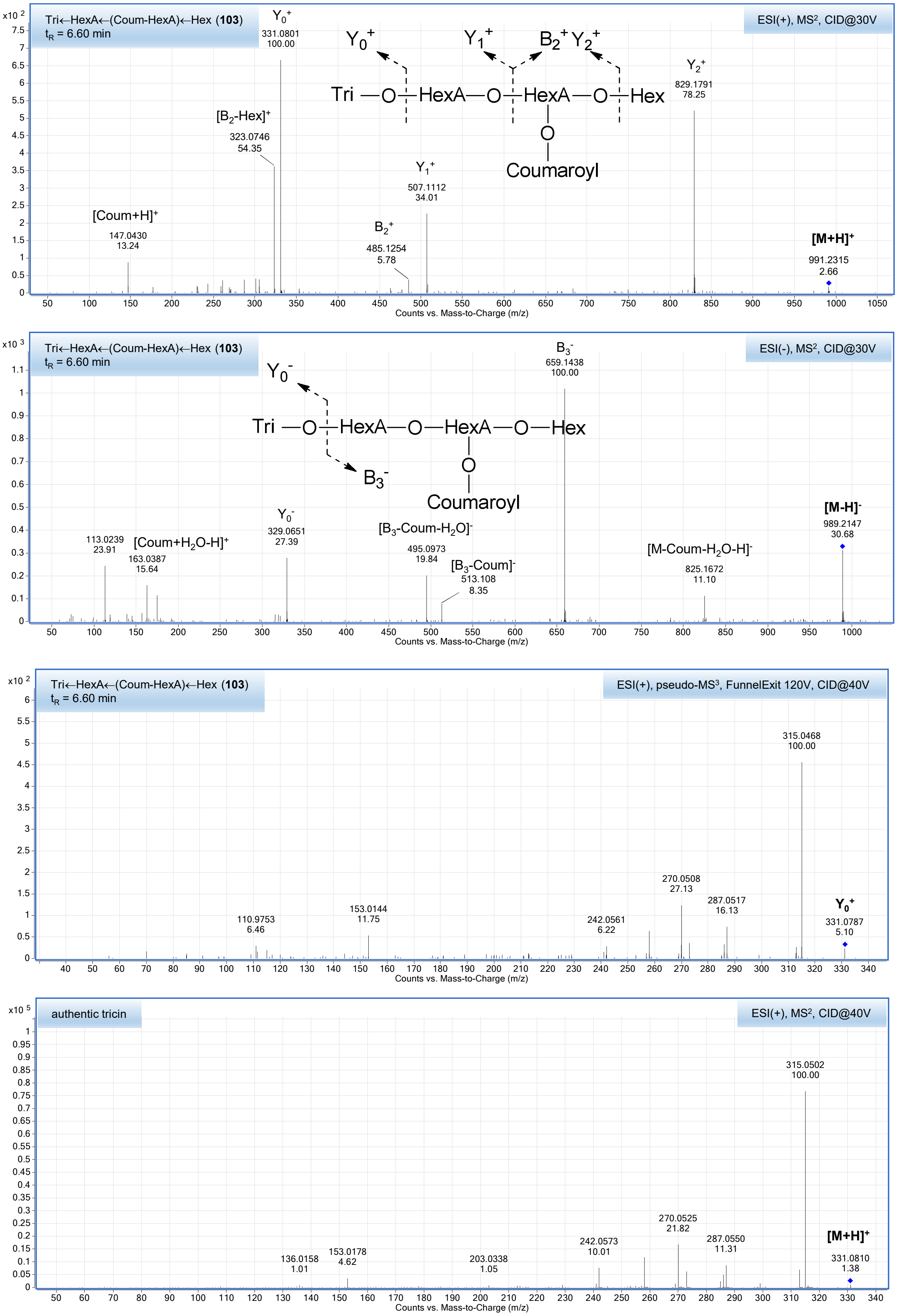

Supplemental Fig. 11 CID mass spectra of the coumaroylated tricin triglycoside **103** detected in a hydromethanolic leaf extract of *A. obliquum* and of authentic tricin. Spectra were obtained using UHPLC/ESI-QTOFMS and chromatographic method B. Precursor ions are marked with a blue diamond.

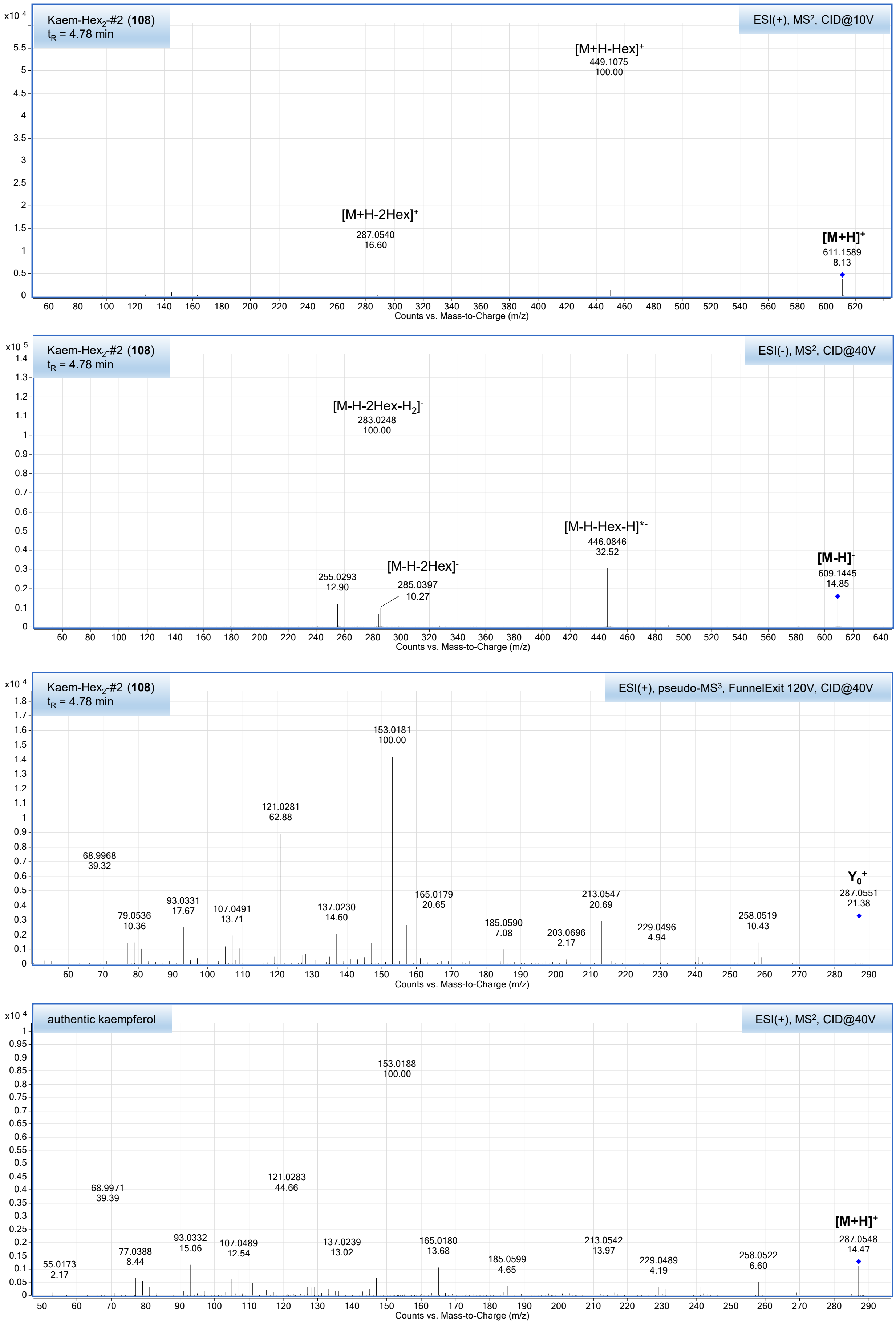

Supplemental Fig. 12 CID mass spectra of the kaempferol di-*O*-hexoside **108** detected in a hydromethanolic leaf extract of *A. obliquum* and of authentic kaempferol. Spectra were obtained using UHPLC/ESI-QTOFMS and chromatographic method B. Precursor ions are marked with a blue diamond.

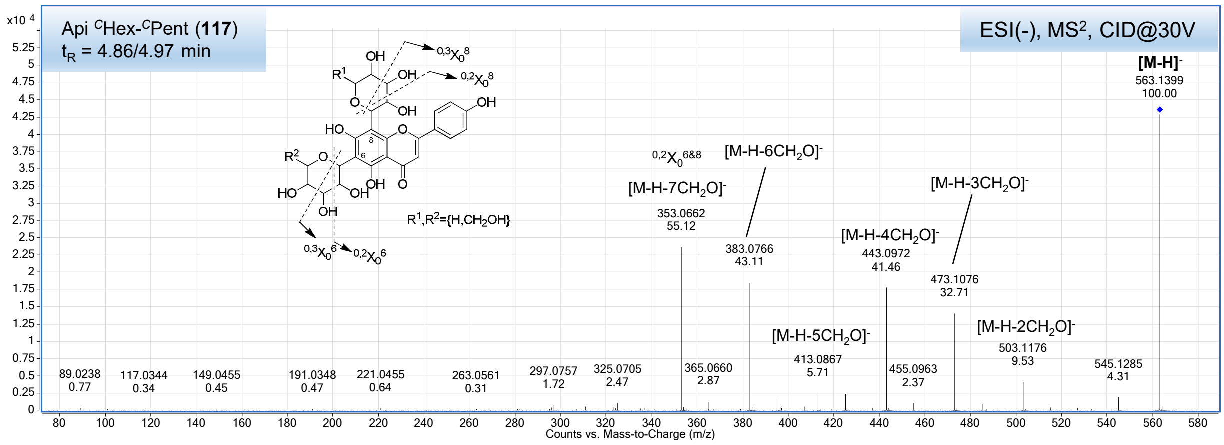

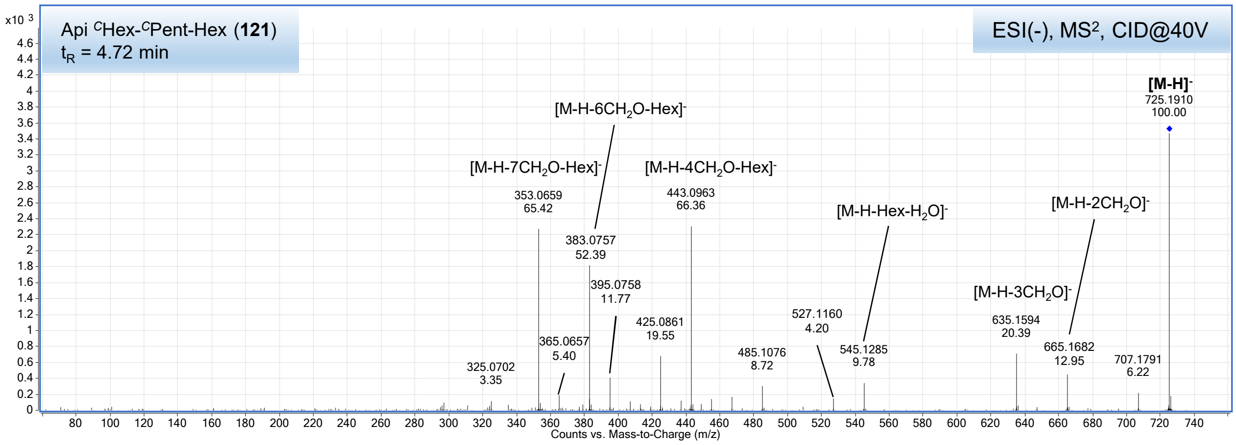

Supplemental Fig. 13 CID mass spectra of the apigenin *C*-glycosides **117** and **121** detected in a hydromethanolic leaf extract of *A. obliquum*. Spectra were obtained using UHPLC/ESI-QTOFMS and chromatographic method B. Precursor ions are bold labelled and marked with a blue diamond.

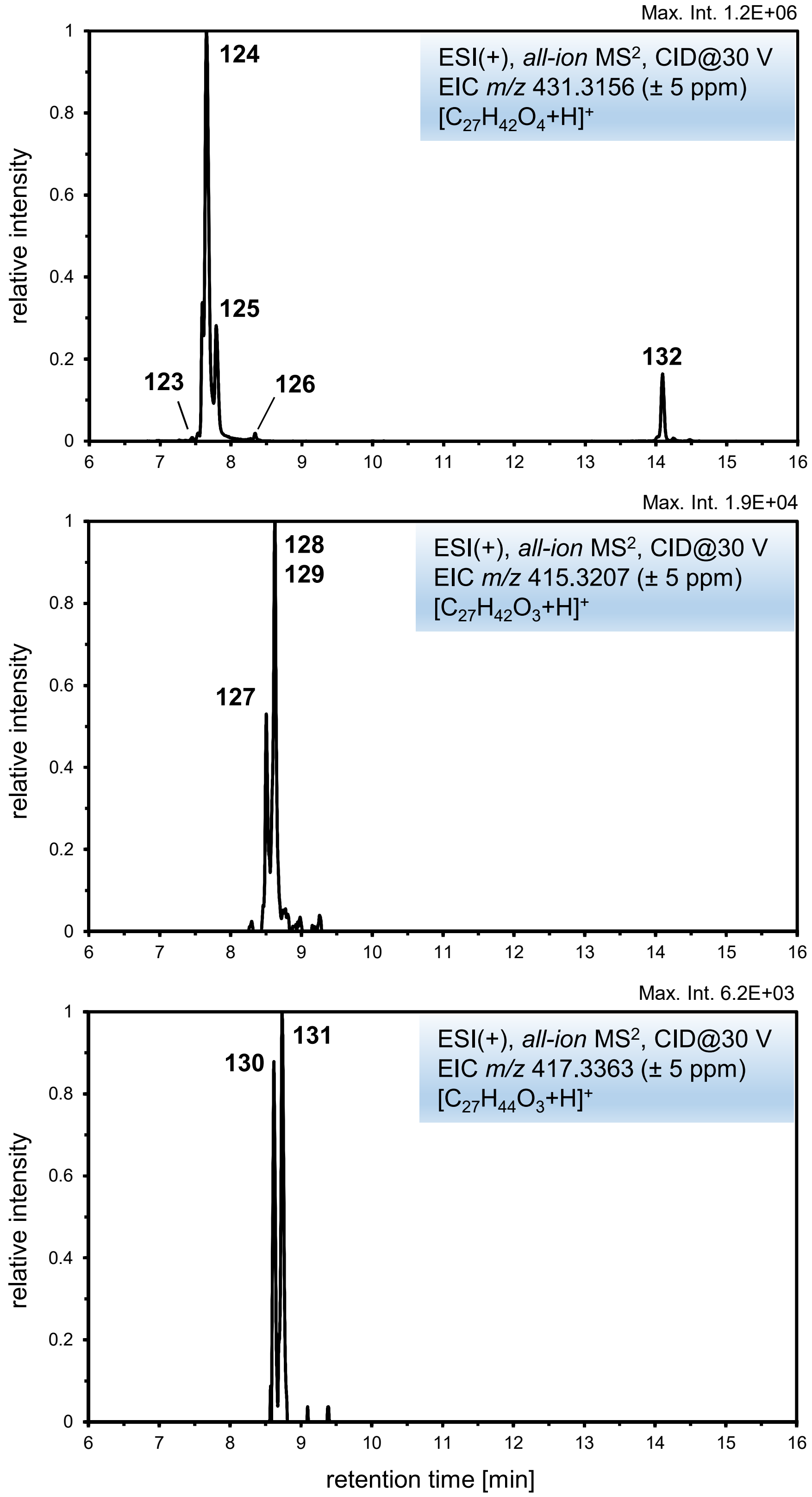

Supplemental Fig. 14 Detection of steroidal saponins in a hydromethanolic bulb extract of *A. obliquum* using all-ion fragmentation. Furostanol saponins **123-131** were detected via their protonated and dehydrated aglycones, the spirostanol saponin **132** via its protonated aglycone. Chromatograms were obtained using UHPLC/ESI-QTOFMS and chromatographic method B in positive ion mode at a collision energy of 30 V.

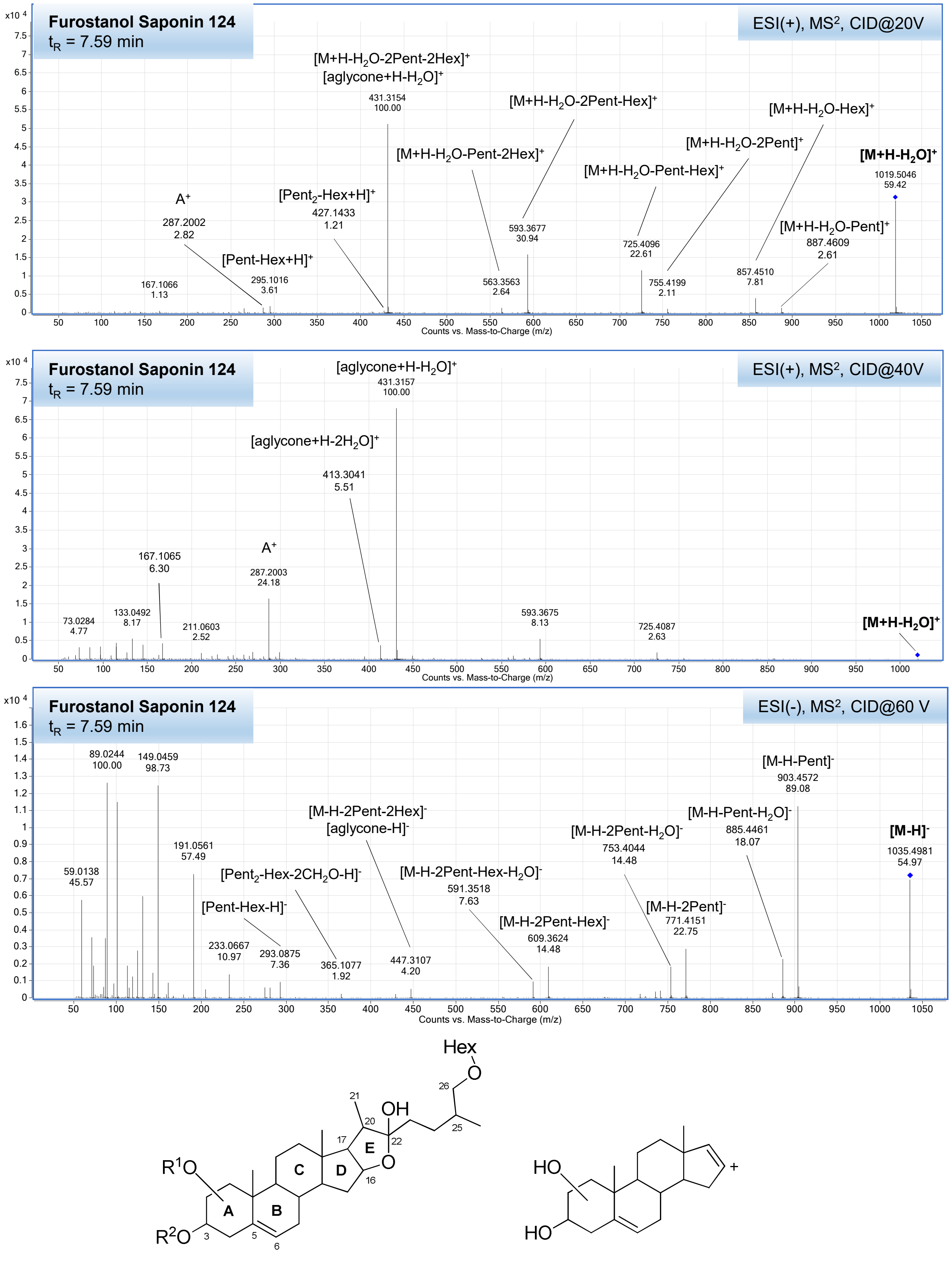

124 R^{1,2}={H, Pent$_2$-Hex)

A⁺

Supplemental Fig. 15 CID mass spectra of the major furostanol saponin **124** detected in a hydromethanolic bulb extract of *A. obliquum*. Spectra were obtained using UHPLC/ESI-QTOFMS and chromatographic method B. Precursor ions are marked with a blue diamond. The positions of the additional hydroxy group and the double bond are unknown.

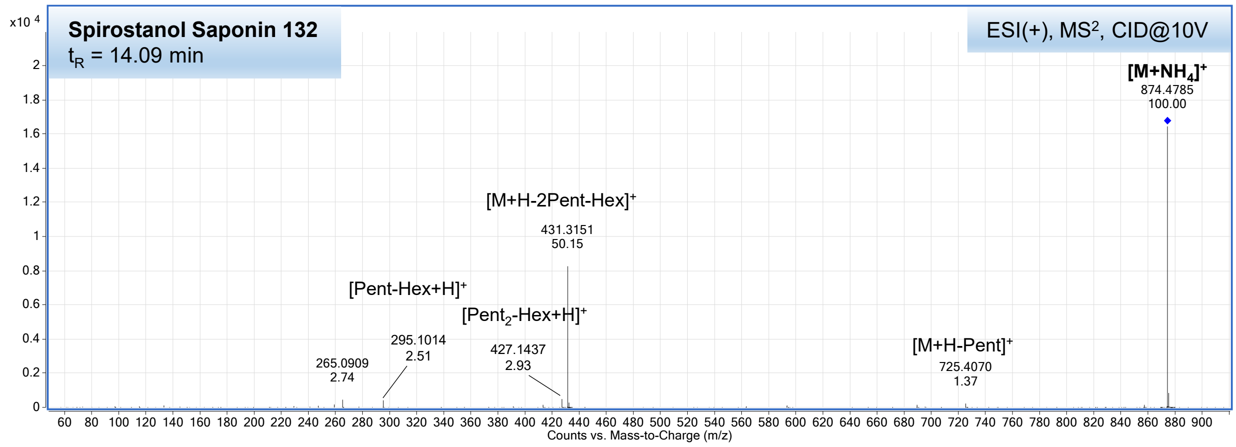

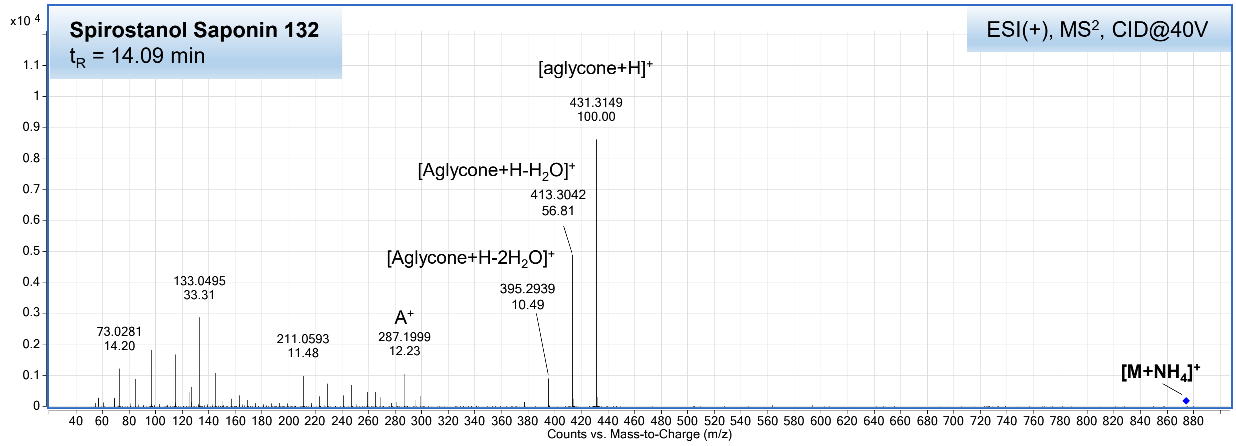

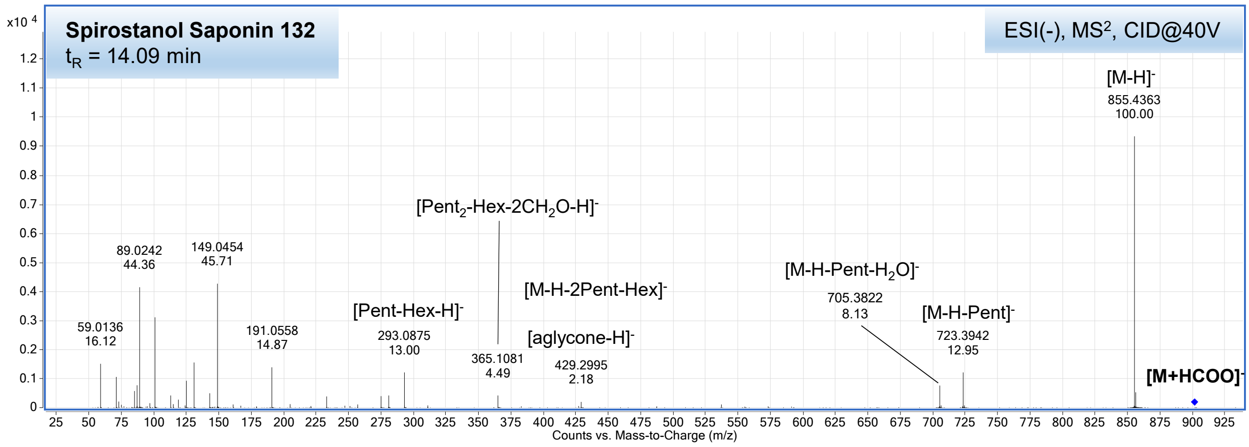

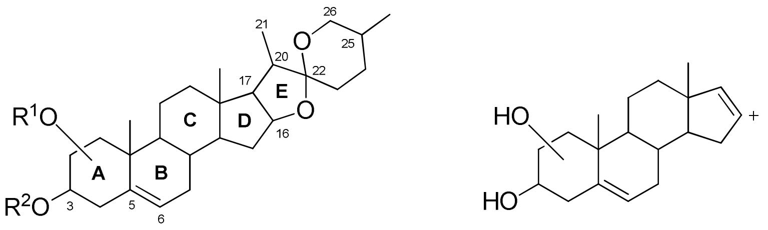

132 $R^{1,2}$={H, $Pent_2$-Hex)

A+

Supplemental Fig. 16 CID mass spectra of the major spirostanol saponin **132** detected in a hydromethanolic bulb extract of *A. obliquum*. Spectra were obtained using UHPLC/ESI-QTOFMS and chromatographic method B. Precursor ions are marked with a blue diamond. The positions of the additional hydroxy group and the double bond are unknown.

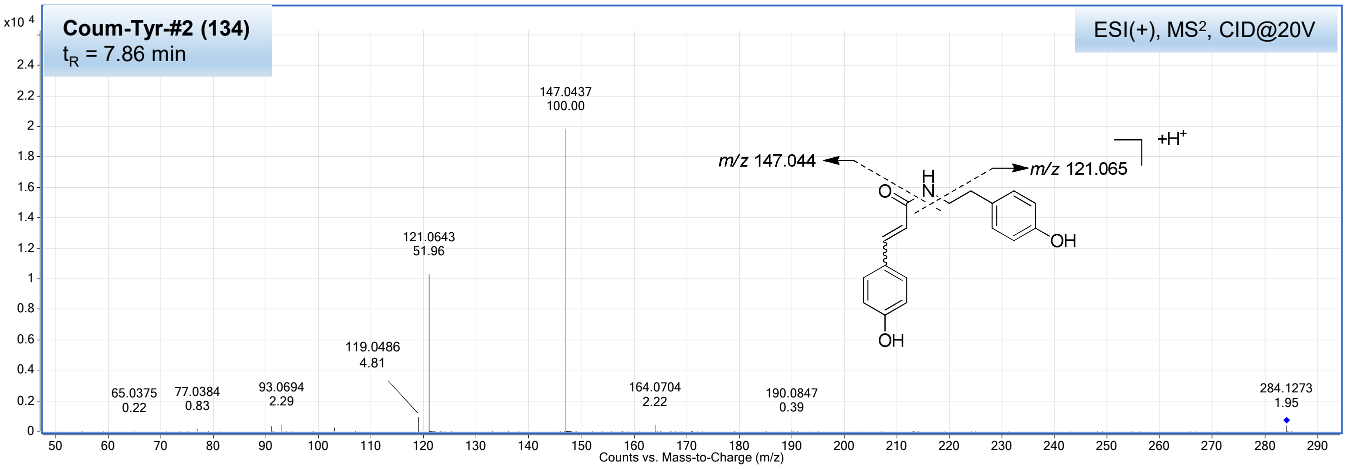

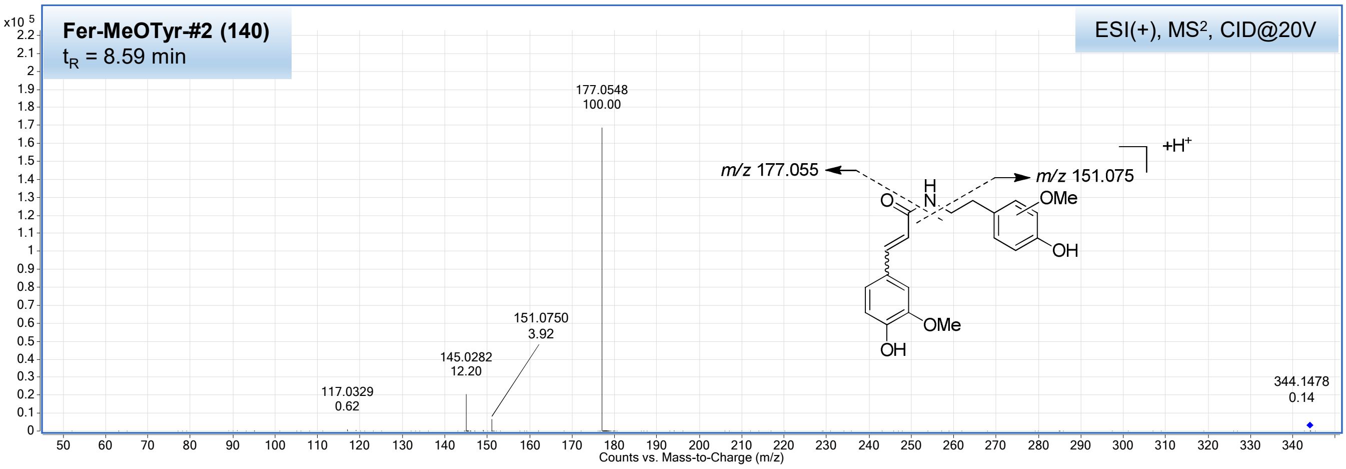

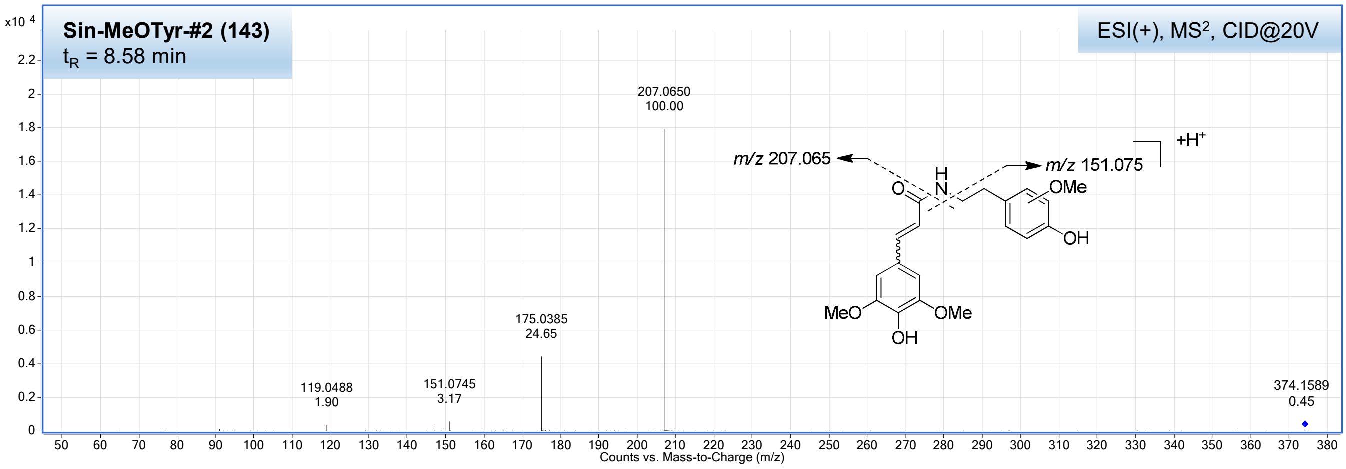

Supplemental Fig. 17 CID mass spectra of the hydroxycinnamic acid amides **134**, **140** and **143** detected in a hydromethanolic leaf extract of *A. obliquum*. Spectra were obtained using UHPLC/ESI-QTOFMS and chromatographic method B. Precursor ions are marked with a blue diamond.

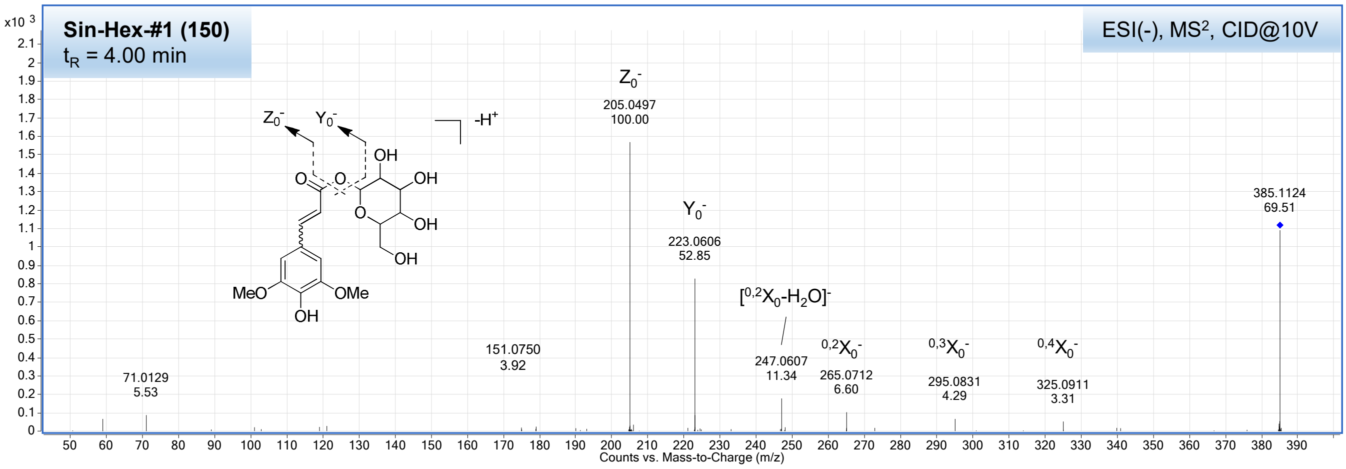

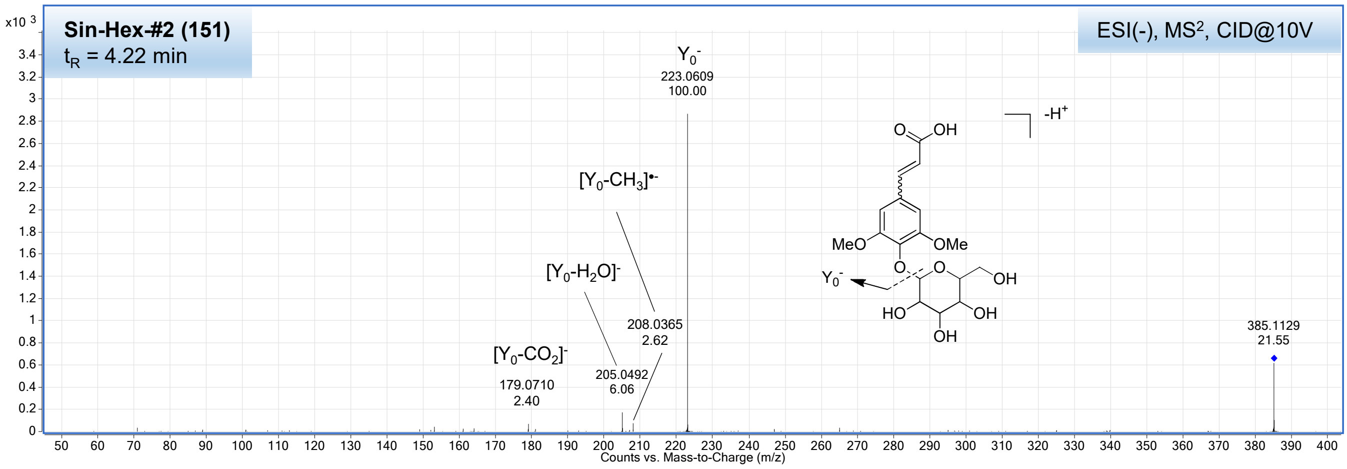

Supplemental Fig. 18 CID mass spectra of the sinapic acid *O*-hexosides **150** and **151** detected in a hydromethanolic leaf extract of *A. obliquum*. Spectra were obtained using UHPLC/ESI-QTOFMS and chromatographic method B. Precursor ions are marked with a blue diamond.

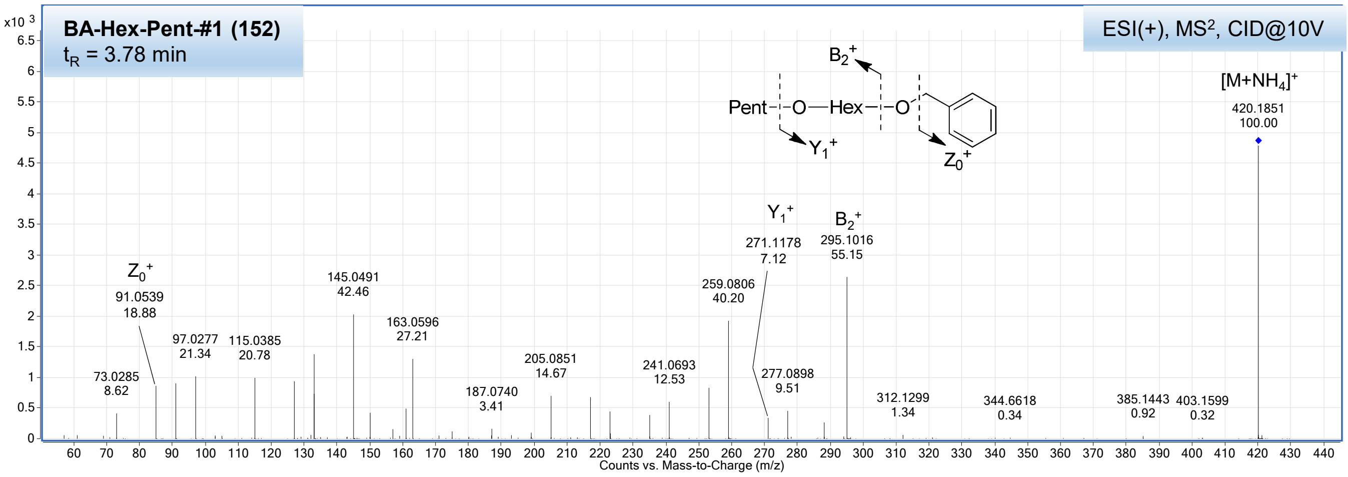

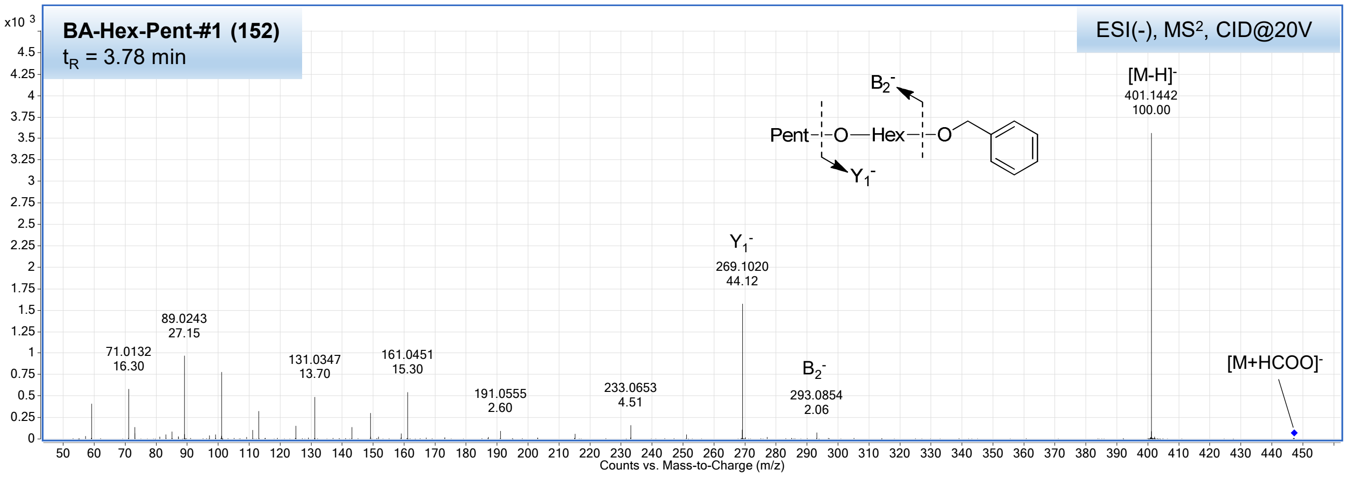

Supplemental Fig. 19 CID mass spectra of the benzylalcohol di-*O*-glycoside **152** detected in a hydromethanolic leaf extract of *A. obliquum*. Spectra were obtained using UHPLC/ESI-QTOFMS and chromatographic method B. Precursor ions are marked with a blue diamond.

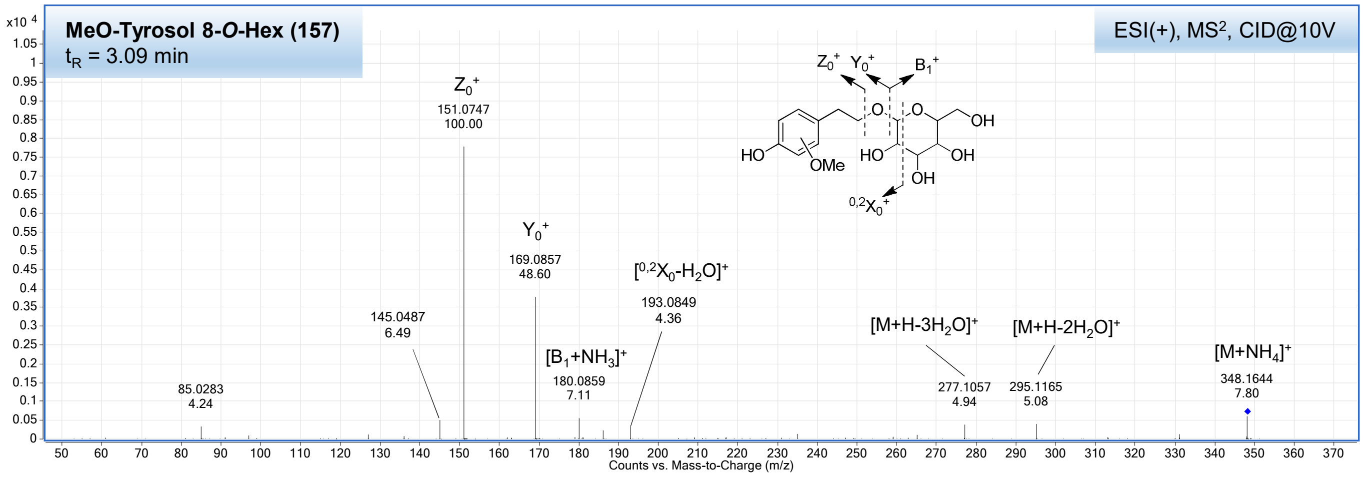

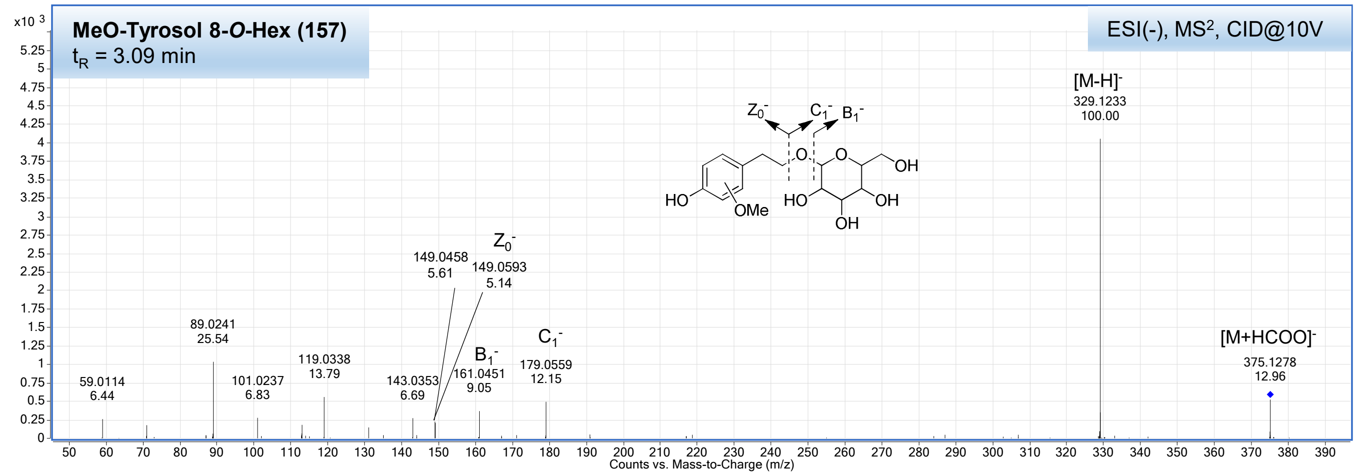

Supplemental Fig. 20 CID mass spectra of the phenylethanoid *O*-hexoside **157** detected in a hydromethanolic leaf extract of *A. obliquum*. Spectra were obtained using UHPLC/ESI-QTOFMS and chromatographic method B. Precursor ions are marked with a blue diamond.

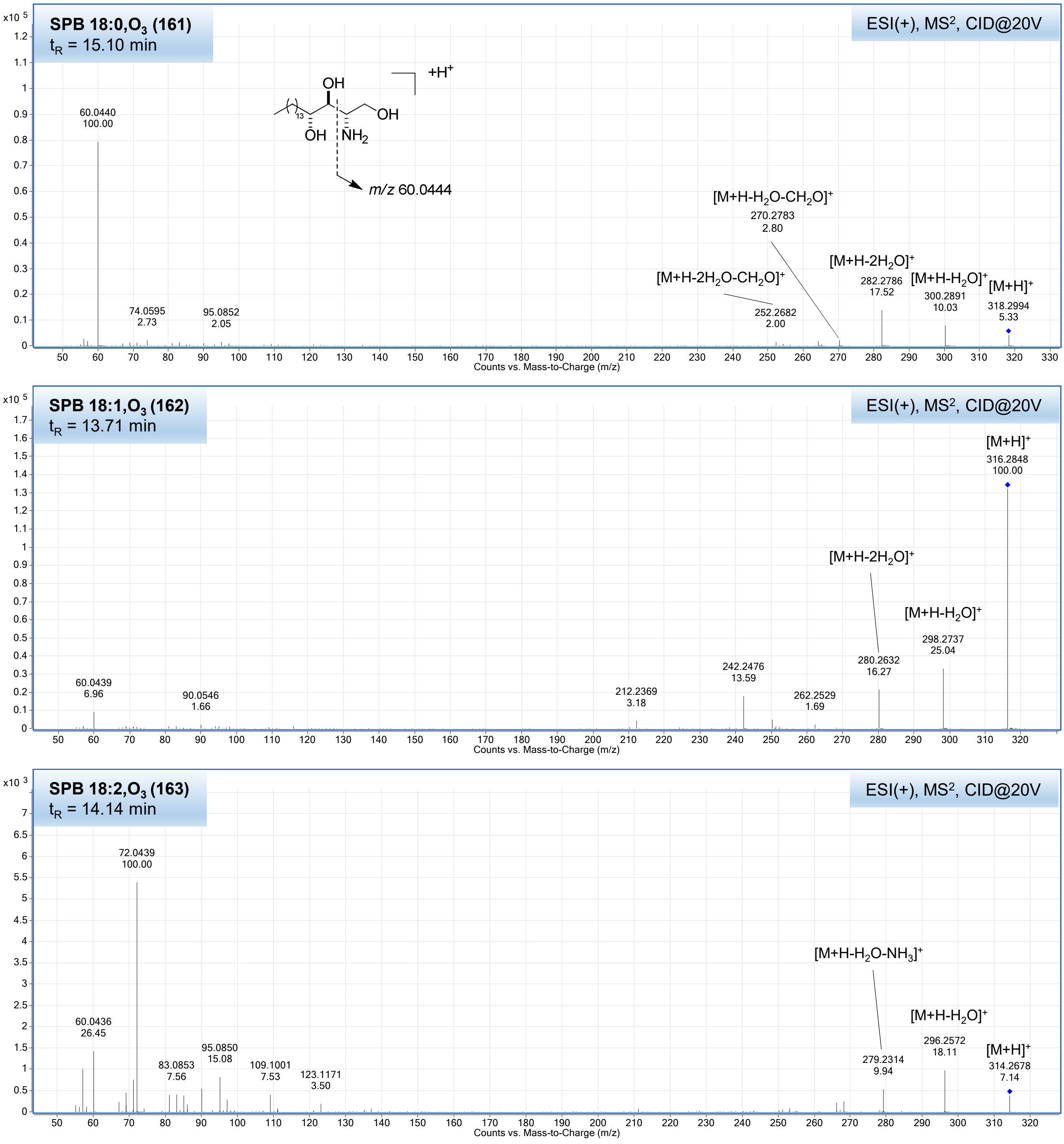

Supplemental Fig. 21 CID mass spectra of sphingoid bases **161**-**163** detected in a hydromethanolic leaf extract of *A. obliquum*. Spectra were obtained using UHPLC/ESI-QTOFMS and chromatographic method B. Precursor ions are marked with a blue diamond. SPB 18:0,O_3 (**161**) was identified as phytosphingosine using a reference compound.

Supplemental Table 1. Reference compounds used for authentication of metabolites and as calibration standards.

No.	Name	CAS	Supplier
-	D-(+)-Glucose	50-99-7	Sigma-Aldrich
-	D-(−)-Fructose	57-48-7	Carl Roth GmbH
-	D-(+)-Sucrose	57-50-1	Carl Roth GmbH
-	Glucuronic acid	6556-12-3	Sigma-Aldrich
-	1-Kestose (1-kestotriose)	470-69-9	Wako Chemicals
-	Nystose (1,1-kestotetraose)	13133-07-8	Wako Chemicals
-	1^F-Fructosylnystose (1,1,1-kestopentaose)	59432-60-9	Wako Chemicals
**1**	(+)-*S*-Methyl-L-cysteine-*S*-oxide ((+)-L-Methiin)	32726-14-0	LKT Labs
**2**	(+)-*S*-Allyl-L-cysteine-*S*-oxide ((+)-L-Alliin)	556-27-4	LKT Labs
**23**	Apigenin	520-36-5	Carl Roth GmbH
**24**	Apigenin 7-*O*-β-D-glucopyranoside	578-74-5	Carl Roth GmbH
**53**	Luteolin	491-70-3	Extrasynthese
**54**	Luteolin 7-*O*-β-D-glucopyranoside	5373-11-5	Carl Roth GmbH
**71**	Chrysoeriol	491-71-4	Extrasynthese
**89**	Tricin	520-32-1	Biosynth-Carbosynth
-	Kaempferol	520-18-3	Indofine
**106**	Kaempferol 3-*O*-β-D-glucopyranoside (Astragalin)	480-10-4	Phytoplan
-	Quercetin dihydrate	6151-25-3	Sigma
**110**	Quercetin 3-*O*-β-D-glucopyranoside (Isoquercitrin)	482-35-9	Phytoplan
-	Isorhamnetin	480-19-3	Carl Roth GmbH
**159**	Tyramine hydrochloride	60-19-5	Sigma-Aldrich
**160**	3-Methoxytyramine hydrochloride	1477-68-5	Sigma-Aldrich
**161**	Phytosphingosine	554-62-1	TCI

Supplemental Table 2. Quantification of non-structural carbohydrates, alk(en)ylcysteine sulfoxides and flavonoid aglycones in bulb and leaf tissue of *A. obliquum*. Means (± ½ range) of two technical replicates are shown. n.d., not detected; FOS, fructooligosaccharides.

Metabolite	Unit	Bulb tissue			Leaf tissue		
		June-2016	*May-2017*	*June-2018*	*June-2016*	*May-2017*	*June-2018*
Fructose	mg (g d.w.)$^{-1}$	44.1±3.0	28.6±0.4	9.7±0.1	67.9±0.7	79.9±1.5	89.7±0.1
Glucose	mg (g d.w.)$^{-1}$	49.7±2.3	76.1±1.5	26.5±0.4	83.1±0.2	94.7±1.4	105±1.8
Sucrose	mg (g d.w.)$^{-1}$	24.8±0.4	49.1±0.3	30.4±0.1	39.8±0.1	47.3±0.5	54.4±0.1
FOS	mg (g d.w.)$^{-1}$	516±2	456±5	660±2	210±5	181±6	162±5
Methiin	mg (g d.w.)$^{-1}$	2.19±0.02	2.85±0.01	4.41±0.01	7.38±0.02	8.62±0.02	7.59±0.01
Alliin	mg (g d.w.)$^{-1}$	3.11±0.07	9.43±0.16	8.62±0.14	5.71±0.09	10.3±0.24	9.62±0.03
Isoalliin	mg (g d.w.)$^{-1}$	1.36±0.04	2.67±0.09	2.98±0.07	1.20±0.03	2.57±0.05	1.92±0.01
Cycloalliin	µg (g d.w.)$^{-1}$	691±34	351±9	847±27	258±7	179±3	333±1
Propiin	µg (g d.w.)$^{-1}$	57±1	74±1	82±2	73±3	72±1	62±1
Apigenin	µg (g d.w.)$^{-1}$	n.d.	n.d.	n.d.	641±14	465±4	751±19
Luteolin	µg (g d.w.)$^{-1}$	n.d.	n.d.	n.d.	617±2	981±2	863±29
Chrysoeriol	µg (g d.w.)$^{-1}$	n.d.	n.d.	n.d.	297±12	276±9	274±7
Selgin	µg (g d.w.)$^{-1}$	n.d.	n.d.	n.d.	30±1	49±1	23±1
Tricin	µg (g d.w.)$^{-1}$	n.d.	n.d.	n.d.	285±5	389±4	274±3
Kaempferol	µg (g d.w.)$^{-1}$	n.d.	n.d.	n.d.	61±5	72±4	81±3
Quercetin	µg (g d.w.)$^{-1}$	n.d.	n.d.	n.d.	34±1	34±1	31±1
Isorhamnetin	µg (g d.w.)$^{-1}$	n.d.	n.d.	n.d.	16±1	14±1	11±1

Supplemental Table 3. Log_2-transformed abundance ratios of annotated metabolites in bulbs and leaves of *A. obliquum*. Metabolites were relatively quantified using peak areas of tabulated quantifier ions given in Supplemental Table 4a-k (average of two technical replicates).

no.	name	log$_2$ [(abundance in bulb)/(abundance in leaf)]		
		June-2016	*May-2017*	*June-2018*
**1**	*S*-Methylcysteine sulfoxide	-1.76	-1.60	-0.78
**2**	*S*-(Prop-2-enyl)cysteine sulfoxide	-0.88	-0.12	-0.16
**3**	*S*-(Prop-1-enyl)cysteine sulfoxide	0.19	0.05	0.63
**4**	3-Methyl-1,4-thiazane-5-carboxylic acid *S*-oxide	1.42	0.97	1.34
**5**	*S*-(Propyl)cysteine sulfoxide	-0.37	0.05	0.40
**6**	*S*-(Methyl)cysteine	-0.50	-0.33	-0.27
**7**	γ-Glutamyl-*S*-(methyl)cysteine	3.43	1.83	3.94
**8**	*S*-(Prop-2-enyl)cysteine	-0.91	0.34	-0.12
**9**	γ-Glutamyl-*S*-(prop-2-enyl)cysteine	3.50	-0.18	3.91
**10**	*S*-(Prop-1-enyl)cysteine	1.07	0.64	0.80
**11**	γ-Glutamyl-*S*-(prop-1-enyl)cysteine	6.73	2.22	6.30
**12**	*S*-(2-Carboxypropyl)cysteine	0.60	-0.55	-0.35
**13**	*S*-(2-Carboxypropyl)cysteinyl-glycine	-2.85	-2.07	-0.39
**14**	*S*-(2-Carboxypropyl)glutathione	-2.45	-1.38	-0.53
**15**	*S*-(Methylthio)cysteine	-2.55	-2.44	-1.54
**16**	γ-Glutamyl-*S*-(methylthio)cysteine	-3.44	-2.24	-3.22
**17**	*S*-(Methylthio)glutathione	-2.80	-0.69	-1.79
**18**	*S*-(Prop-2-enylthio)cysteine	-1.68	-1.25	-1.03
**19**	γ-Glutamyl-S-(prop-2-enylthio)cysteine	-1.99	-0.32	-1.77
**20**	*S*-(Prop-2-enylthio)glutathione	-1.44	0.77	-1.80
**21**	*S*-(Prop-1-enylthio)cysteine	-1.28	-1.20	-0.34
**22**	*S*-(Prop-1-enylthio)glutathione	-0.73	-0.24	-1.31
**23-52**	Apigenin *O*-Glycosides	n.d. in bulbs	n.d. in bulbs	n.d. in bulbs
**53-70**	Luteolin *O*-Glycosides	n.d. in bulbs	n.d. in bulbs	n.d. in bulbs
**71-82**	Chrysoeriol *O*-Glycosides	n.d. in bulbs	n.d. in bulbs	n.d. in bulbs
**83-88**	Selgin *O*-Glycosides	n.d. in bulbs	n.d. in bulbs	n.d. in bulbs
**89-105**	Tricin *O*-Glycosides	n.d. in bulbs	n.d. in bulbs	n.d. in bulbs
**106-115**	Flavonol *O*-Glycosides	n.d. in bulbs	n.d. in bulbs	n.d. in bulbs
**116-122**	Flavone *C*-Glycosides	n.d. in bulbs	n.d. in bulbs	n.d. in bulbs
**123**	$C_{27}H_{44}O_5$ Hex$_3$-Pent	n.d. in leaves	n.d. in leaves	n.d. in leaves
**124**	$C_{27}H_{44}O_5$ Hex$_2$-Pent$_2$	8.73	5.54	4.89
**125**	$C_{27}H_{44}O_5$ Hex$_2$-Pent	n.d. in leaves	5.05	5.27
**126**	$C_{27}H_{44}O_5$ Hex$_2$	n.d. in leaves	5.93	6.84
**127**	$C_{27}H_{44}O_4$ Hex$_5$	n.d. in leaves	5.04	5.99
**128**	$C_{27}H_{44}O_4$ Hex$_4$	n.d. in leaves	n.d. in leaves	n.d. in leaves
**129**	$C_{27}H_{44}O_4$ Hex$_2$-Pent$_2$	n.d. in leaves	4.99	4.62
**130**	$C_{27}H_{46}O_4$ Hex$_5$	n.d. in leaves	5.65	5.66
**131**	$C_{27}H_{46}O_4$ Hex$_4$	n.d. in leaves	n.d. in leaves	n.d. in leaves
**132**	$C_{27}H_{42}O_4$ Hex-Pent$_2$	9.34	4.34	1.87
**133**	*N*-Coumaroyl-tyramine - isomer#1	-0.07	-1.08	-4.31
**134**	*N*-Coumaroyl-tyramine - isomer#2	-1.04	-1.20	-3.05
**135**	*N*-Coumaroyl-methoxytyramine - isomer#1	1.00	0.76	-1.32
**136**	*N*-Coumaroyl-methoxytyramine - isomer#2	-2.20	-2.37	-3.23
**137**	*N*-Feruloyl-tyramine - isomer#1	0.97	0.71	-1.33
**138**	*N*-Feruloyl-tyramine - isomer#2	-2.20	-2.37	-3.23
**139**	*N*-Feruloyl-methoxytyramine - isomer#1	-1.79	-1.99	-3.26
**140**	*N*-Feruloyl-methoxytyramine - isomer#2	-3.12	-3.31	-4.09
**141**	*N*-Sinapoyl-tyramine	-3.12	-3.31	-4.09
**142**	*N*-Sinapoyl-methoxytyramine - isomer#1	-0.72	-0.23	1.34
**143**	*N*-Sinapoyl-methoxytyramine - isomer#2	-3.29	-2.70	-0.05
**144**	1-*O*-Coumaroyl hexose - isomer#1	-3.57	-3.73	-4.28
**145**	1-*O*-Coumaroyl hexose - isomer#2	n.d. in bulbs	n.d. in bulbs	n.d. in bulbs
**146**	1-*O*-Feruloyl hexose - isomer#1	-5.83	-4.72	-4.33
**147**	1-*O*-Feruloyl hexose - isomer#2	-4.98	-3.95	-3.70
**148**	Ferulic acid 4-*O*-(*O*-hexosyl-hexoside) - isomer#1	-3.21	-1.54	-1.06
**149**	Ferulic acid 4-*O*-(*O*-hexosyl-hexoside) - isomer#2	-0.54	-1.38	-1.87
**150**	1-*O*-Sinapoyl hexose	-1.44	0.19	0.37
**151**	Sinapic acid 4-*O*-hexoside	-1.47	-1.60	-2.17

**152**	Benzylalcohol *O*-(*O*-pentosyl-hexoside) -isomer#1	n.d. in bulbs	n.d. in bulbs	n.d. in bulbs
**153**	Benzylalcohol *O*-(*O*-pentosyl-hexoside) -isomer#2	n.d. in bulbs	n.d. in bulbs	n.d. in bulbs
**154**	Phenylethanol *O*-(*O*-pentosyl-hexoside) -isomer#1	n.d. in bulbs	n.d. in bulbs	n.d. in bulbs
**155**	Phenylethanol *O*-(*O*-pentosyl-hexoside) -isomer#2	n.d. in bulbs	n.d. in bulbs	n.d. in bulbs
**156**	Tyrosol 8-*O*-hexoside	-4.31	-5.06	-6.86
**157**	Methoxytyrosol 8-*O*-hexoside	-5.42	-5.84	-7.39
**158**	Methoxytyrosol *O*-(*O*-pentosyl-hexoside)	n.d. in bulbs	n.d. in bulbs	n.d. in bulbs
**159**	Tyramine	-2.25	-2.56	-2.93
**160**	3-Methoxytyramine	-2.09	-1.67	-1.04
**161**	SPB 18:0,O_3 (Phytosphingosine)	-0.71	-1.30	-2.11
**162**	SPB 18:1,O_3	-1.80	-2.46	-5.06
**163**	SPB 18:2,O_3	-0.62	-1.73	-5.18
**164**	SPB 18:0,O_4	n.d. in bulbs	n.d. in bulbs	n.d. in bulbs
**165**	SPB 18:1,O_4- isomer #1	-1.22	-2.32	-4.29
**166**	SPB 18:1,O_4- isomer #2	-0.30	-0.94	-2.62
**167**	SPB 18:2,O_4	-0.86	-2.31	-4.11

Supplemental Method 1. Isolation and structural characterization of flavone glycosides

Freeze-dried and homogenized leaf material (25 g) was sequentially extracted with n-hexane (2 × 250 ml), ethyl acetate (2 × 250 ml) and methanol/water, 4/1 (v/v) (2 × 250 ml). The combined hydromethanolic extracts were evaporated to dryness at 40 °C *in vacuo.* The resulting raw extract (10.5 g) was solubilized in 25 ml methanol/water, 5/95 (v/v) and fractionated using a column (30 × 270 mm) packed with Diaion HP-20 polymeric adsorbent. The column was consecutively eluted with methanol/water, 5/95 (v/v, 500 mL, fraction 1, yield 10.2 g), 15/85 (v/v, 500 mL, fraction 2, yield 0.05 g), 30/70 (v/v, 500 mL, fraction 3, yield 0.06 g), 45/55 (v/v, 500 mL, fraction 4, 0.08 g) and methanol (500 mL, fraction 5, 0.32 g). Fraction 1 - 5 were analyzed by LC/MS (chromatographic method B). For isolation of flavone glycosides fraction 3 and 4 were evaporated to dryness at 40 °C *in vacuo,* dissolved each in 2 mL methanol/water, 1/1 (v/v) and subjected to semi-preparative HPLC. Semi-preparative HPLC was performed on an Agilent 1100 Series HPLC system equipped with a ReproSil-XR 120 C18 column (8 × 250 mm, particle size 5 µm, Dr. Maisch) at 40 °C. Water/acetonitrile, 95/5 (v/v, A) and water/acetonitrile, 45/55 (v/v, B) were used as eluents. The following binary gradient program at a flow rate of 2.5 mL min^{-1} was applied: 0–5 min, isocratic 0 % B, 5–35 min, linear from 0 % to 18 % B. The injection volume was 30 µL. Compounds were monitored by a photodiode array detector at 254 nm and 340 nm and collected manually. This afforded 9.3 mg **32**, 2.5 mg **58** and 0.5 mg **92** which were characterized by NMR spectroscopy and sugar analysis.

One- (^1H, ^{13}C) and two-dimensional (^1H-^1H-COSY, ^1H-^{13}C-HMQC, ^1H-^{13}C-HMBC) NMR spectra were recorded using a Bruker AVANCE III NMR spectrometer (^1H and ^{13}C resonance frequencies 700 MHz and 176 MHz) equipped with a 5 mm He cryo probe at 298.15 K. All spectra were referenced to dimethylsulfoxide-D_6 at δH 2.50 ppm and δC 39.5 ppm, respectively.

For acidic hydrolysis, 50 µg of the flavone glycoside were incubated in 200 µL conc. hydrochloric acid/acetic acid/water, 10/35/55 (v/v/v) under shaking (2 h, 990 min^{-1}, 90 °C). After cooling to room temperature, 10 µl of the reaction mixture were transferred into a GC vial and evaporated to dryness using a centrifugal vacuum concentrator (35 °C, 10 mbar). Released monosaccharides were in-line derivatized with methoxyamine and *N*-methyl-*N*-(trimethylsilyl)trifluoroacetamide and analyzed by GC/MS according to a standard protocol (Erban et al., 2007).

Apigenin 7-*O*-β-(2´´-*O*-β-glucuronopyranosyl)-glucopyranoside (32)

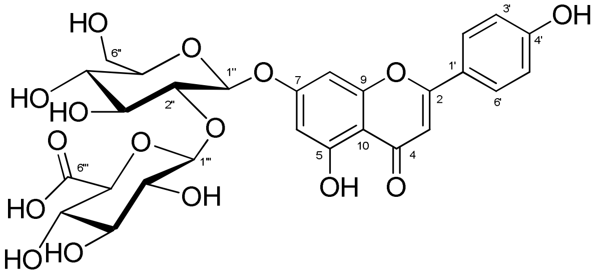

Analysis of the derivatized acid hydrolysate of **32** by GC/MS revealed the presence of glucose and glucuronic acid. Protonated **32** (*m/z* 609.145) formed Y_1^+ at *m/z* 433.111 and Y_0^+ ions at *m/z* 271.060, deprotonated **32** (*m/z* 607.130) B_2^- ions at *m/z* 337.077 and Y_0^- ions at *m/z* 269.048, indicating the general structure apigenin ← Glc ← GlcA. In the ^{1}H NMR spectrum of **32**, resonances of H-6 (δ_H 6.49) and H-8 (δ_H 6.90) are downfield shifted compared to apigenin (H-6: δ_H 6.25, H-8: δ_H 6.52), suggesting glycosylation of the aglycone in position *O*-7 (Markham et al., 1994). The anomeric protons of the two sugar residues appear as doublets at δ_H 5.18 (J = 7.5 Hz, δ_C 98.3) and δ_H 4.54 (J = 7.9 Hz, δ_C 104.0) with $J_{H1,H2}$ coupling constants indicating a β configuration of both anomeric carbon atoms (Markham et al., 1994). A down-field shift characteristic for glycosylation was registered for Glc H-2´´ (δ_H 3.50) and C-2´´ (δ_C 82.4). Long-range ^{1}H-^{13}C connectivities were detected in the HMBC spectrum for Glc H-1´´ (δ_H 5.18) and apigenin C-7 (δ_C 162.9) and for GlcA H-1´´´ (δ_H 4.54) and Glc C-2´´ (δ_C 82.4).

^{1}H NMR (700 MHz, DMSO-D_6): δ 3.04 (dd, 8.0 Hz, 8.0 Hz, H-2´´´), 3.19-3.26 (m, H-4´´, H-3´´´, H-4´´´), 3.47-3.57 (m, H-2´´, H-3´´, H-5´´, H_a-6´´, H-5´´), 3.73 (dd, 10.5 Hz, 4.5 Hz, H_b-6´´), 4.54 (d, 7.9 Hz, H-1´´´), 5.18 (d, 7.5 Hz, H-1´´), 6.49 (br. s, H-6), 6.84 (s, H-3), 6.90 (br. S, H-8), 6.93 (d, 8.5 Hz, H-3´, H-5´), 7.95 (d, 8.5 Hz, H-2´, H-6´).

^{13}C NMR (176 MHz, DMSO-D_6): δ 60.5 (C-6´´), 69.1 (C-4´´), 71.9 (C-4´´´), 74.3 (C-2´´´), 75.0 (C-3´´), 75.6 (C-5´´´), 76.0 (C-3´´´), 77.0 (C-5´´), 82.4 (C-2´´), 95.6 (C-8), 98.3 (C-1´´), 99.8 (C-6), 103.0 (C-3), 104.0 (C-1´´´), 105.4 (C-10), 116.0 (C-3´,C-5´), 121.0 (C-1´), 128.6 (C-2´, C-6´), 156.8 (C-9), 161.1 (C-5), 161.4 (C-4´), 162.9 (C-7), 164.3 (C-2), 171.2 (C-6´´´), 182.1 (C-4).

Luteolin 7-*O*-β-(2´´-O- β-glucuronopyranosyl)-glucopyranoside (58)

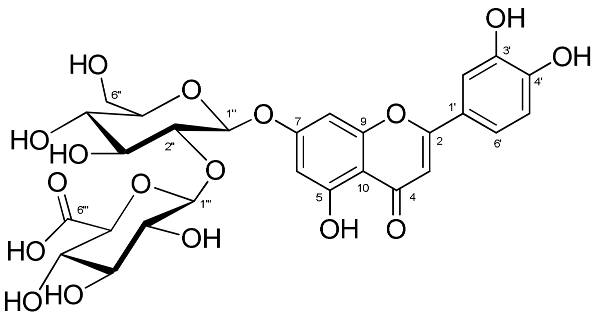

Analysis of the derivatized acid hydrolysate of **58** by GC/MS revealed the presence of glucose and glucuronic acid. Protonated **58** (*m/z* 625.139) formed Y_1^+ at *m/z* 449.107 and Y_0^+ ions at *m/z* 287.055, deprotonated **58** (*m/z* 623.125) B_2^- ions at *m/z* 337.077 and Y_0^- ions at *m/z* 285.040, indicating the general structure luteolin ← Glc ← GlcA. In the ^{1}H NMR spectrum of **58**, resonances of H-6 (δ_H 6.48) and H-8 (δ_H 7.01) are downfield shifted compared to luteolin (H-6: δ_H 6.22, H-8: δ_H 6.47), suggesting glycosylation of the aglycone in position *O*-7 (Markham et al., 1994). The anomeric protons of the two sugar residues appear as doublets at δ_H 5.21 (J = 7.5 Hz, δ_C 98.9) and δ_H 4.53 (J = 7.8 Hz, δ_C 104.1) with $J_{H1,H2}$ coupling constants indicating a β configuration of both anomeric carbon atoms (Markham et al., 1994). A down-field shift characteristic for glycosylation was registered for Glc H-2´´ (δ_H 3.50) and C-2´´ (δ_C 82.5). Long-range ^{1}H-^{13}C connectivities were detected in the HMBC spectrum for Glc H-1´´ (δ_H 5.21) and apigenin C-7 (δ_C 162.8) and for GlcA H-1´´´ (δ_H 4.53) and Glc C-2´´ (δ_C 82.5).

^{1}H NMR (DMSO-D_6): δ 3.04 (dd, 8.0 Hz, 8.0 Hz, H-2´´´), 3.19-3.26 (m, H-4´´, H-3´´´, H-4´´´), 3.47-3.57 (m, H-2´´, H-3´´, H-5´´, H_a-6´´, H-5´´), 3.73 (dd, 10.4 Hz, 4.5 Hz, H_b-6´´), 4.53 (d, 7.8 Hz, H-1´´´), 5.21 (d, 7.5 Hz, H-1´´), 6.48 (br. s, H-6), 6.72 (s, H-3), 6.87 (d, 8.3 Hz, H-5´), 7.01 (br. s, H-8), 7.42 (dd, 8.4 Hz, 2.3 Hz, H-6´), 7.64 (br. s, H-2´).

^{13}C NMR (DMSO-D_6): δ 60.7 (C-6´´), 69.2 (C-4´´), 71.9 (C-4´´´), 74.4 (C-2´´´), 74.8 (C-3´´), 75.6 (C-5´´´), 76.1 (C-3´´´), 77.0 (C-5´´), 82.5 (C-2´´), 96.3 (C-8), 98.9 (C-1´´), 100.1 (C-6), 102.8 (C-3), 104.1 (C-1´´´), 105.5 (C-10), 114.1 (C-2´), 116.0 (C-5´), 118.8 (C-6´), 121.3 (C-1´), 146.0 (C-3´), 149.9 (C-4´), 156.7 (C-9), 160.9 (C-5), 162.8 (C-7), 164.5 (C-2), 171.3 (C-6´´´), 182.0 (C-4).

Tricin 7-*O*-β-glucuronopyranoside (92)

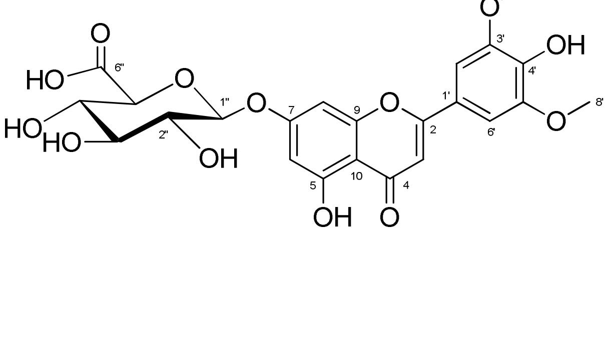

Analysis of the derivatized acid hydrolysate of **92** by GC/MS revealed the presence of glucuronic acid. Protonated **92** (*m/z* 507.144) formed Y_0^+ ions at *m/z* 331.081, deprotonated **92** (*m/z* 505.099) Y_0^- ions at *m/z* 329.067, indicating the general structure tricin ← GlcA. In the ^{1}H NMR spectrum of **92**, resonances of H-6 (δ_H 6.47) and H-8 (δ_H 6.92) are downfield shifted compared to tricin (H-6: δ_H 6.21, H-8: δ_H 6.56), suggesting glycosylation of the aglycone in position *O*-7 (Markham et al., 1994). The anomeric proton of the sugar residue appears as doublet at δ_H 5.16 (J = 7.5 Hz, δ_C 99.4) with a $J_{H1,H2}$ coupling constant indicating a β configuration of the anomeric carbon atom (Markham et al., 1994).

^{1}H NMR (DMSO-D_6): δ 3.25-3.40 (m, H-2´´, H-3´´, H-4´´, interference with residual water signal), 3.89 (m, H-7´, H-8´, H-5´), 5.16 (d, 7.5 Hz, H-1´´), 6.47 (d, 2.2 Hz, H-6), 6.92 (d, 2.2 Hz, H-8), 7.07 (s, H-3), 7.36 (s, H-2´, H-6´).

^{13}C NMR (DMSO-D_6): δ 56.4 (C-7´, C-8´), 72.5 (C-2´´), 75.0 (C-5´´), 76.1 (C-3´´), 95.0 (C-8), 99.4 (C-6), 99.4 (C-1´´), 103.8 (C-3), 104.6 (C-2´, C-6´), 105.3 (C-10), 120.2 (C-1´), 140.1 (C-4´), 148.2 (C-3´, C-5´), 156.9 (C-9), 161.2 (C-5), 162.9 (C-7), 164.2 (C-2), 182.0 (C-4), ^{13}C chemical shifts were obtained from HMQC and HMBC spectra, chemical shifts of C-4´´ and C-6´´ could not be extracted. Full NMR data are given in the literature (Stochmal et al., 2001).

Erban, A., Schauer, N., Fernie, A. R., & Kopka, J. (2007). Nonsupervised construction and application of mass spectral and retention time index libraries from time-of-flight gas chromatography-mass spectrometry metabolite profiles. *Methods in Molecular Biology, 358*, 19-38. https://doi.org/10.1007/978-1-59745-244-1_2.

Markham, K. R., & Geiger, H. (1994). ^{1}H NMR spectroscopy of flavonoids and their glycosides in DMSO-d6. In J. B. Harborne (Ed.), *The Flavonoids: Advances in Research Since 1986* (pp. 441-497). London: Chapman and Hall.

Stochmal, A., Simonet, A. M., Macias, F. A., & Oleszek, W. (2001). Alfalfa (*Medicago sativa* L.) flavonoids. 2. Tricin and chrysoeriol glycosides from aerial parts. *Journal of Agricultural and Food Chemistry, 49*(11), 5310-5314. https://doi.org/10.1021/jf010600x.

Supplemental Method 2. Isolation and enzymatic hydrolysis of the major furostanol saponin **124**

Freeze-dried and homogenized bulb material (1 g) was extracted with methanol/water, 4/1 (v/v) (2 × 10 ml). The combined hydromethanolic extracts were evaporated to dryness at 40 °C *in vacuo.* The resulting residue was solubilized in 1 ml methanol/water, 50/50 (v/v) and subjected to semi-preparative HPLC. Semi-preparative HPLC was performed on an Agilent 1100 Series HPLC system equipped with a ReproSil-XR 120 C18 column (8 × 250 mm, particle size 5 µm, Dr. Maisch) at 40 °C. Water (A) and acetonitrile (B) were used as eluents. The following binary gradient program at a flow rate of 3 mL min^{-1} was applied: 0–5 min, isocratic 5 % B, 5–25 min, linear from 5 % to 45 % B. The injection volume was 50 µL. Fractions were collected from 6 to 25 min every 30 s and screened for saponin **124** by UHPLC/ESI-QTOFMS (chromatographic method B, positive ion mode). Fractions from five separations containing saponin **124** were combined. An aliquot (500 µL) of the combined fractions was transferred into a 2-mL microcentrifuge tube and evaporated to dryness using a centrifugal vacuum concentrator (35 °C, 10 mbar). The remaining residue was redissolved in 200 µL sodium acetate buffer (50 mM, pH 5.0). After addition of 100 µL freshly prepared β-glucosidase solution (0.134 units $µL^{-1}$, in 50 mM sodium acetate buffer, pH 5.0) the mixture was shaken (24 h, 990 min^{-1}, 40 °C). Afterwards, the mixture was extracted three times with 400 µL ethyl acetate. The organic extracts were combined and evaporated to dryness using a centrifugal vacuum concentrator (35 °C, 10 mbar). The remaining residue was redissolved in 500 µL methanol and analyzed by UHPLC/ESI-QTOFMS using chromatographic method B and the positive ion mode.
